# MicroRNA-21 promotes dysregulated lipid metabolism and hepatocellular carcinoma

**DOI:** 10.1242/dmm.052583

**Published:** 2026-03-05

**Authors:** Chad VanSant-Webb, Jessye C. Castro, Audrey Y. Su, Kiandra Hawkins, Aavrati Saxena, Jillian Wright, Richard Smith, Marco Fragoso-García, Yian Ann Chen, Carrie Barton, Chris Stubben, Ryan M. O'Connell, Gregory S. Ducker, Kimberley J. Evason

**Affiliations:** ^1^ Department of Pathology, University of Utah, 15 North Medical Drive East, Salt Lake City, UT 84101, USA; ^2^ Huntsman Cancer Institute, 2000 Circle of Hope Way, Salt Lake City, UT 84101, USA; ^3^ Department of Biochemistry, University of Utah, 15 N Medical Drive E, Salt Lake City, UT 84112, USA; ^4^ Department of Pathology, Albert Einstein College of Medicine, 1301 Morris Park Avenue, Bronx, NY 10461, USA; ^5^ Department of Population Health Sciences, University of Utah, 295 Chipeta Way, Salt Lake City, UT 84108, USA; ^6^ Department of Internal Medicine, University of Utah, 30 N Mario Capecchi Dr., Salt Lake City, UT 84112, USA; ^7^ Office of Comparative Medicine, 50 S 2030 E, Salt Lake City, UT 84132, USA

**Keywords:** Zebrafish, Acylcarnitines, Liver, β-catenin, Steatosis

## Abstract

The prevalence of hepatocellular carcinoma (HCC) is rising in parallel with increasing obesity and metabolic dysfunction-associated steatohepatitis (MASH). MicroRNAs (miRNAs) are key post-transcriptional regulators of gene expression and attractive targets for HCC therapy. Here, we sought to identify and characterize dysregulated miRNAs in MASH-driven HCC (MASH-HCC). We profiled miRNA expression in liver tissue from patients with MASH or MASH-HCC and in zebrafish HCC driven by activated β-catenin (CTNNB1), one of the most commonly mutated oncogenes in MASH-HCC. We found overlap between dysregulated human and zebrafish miRNAs, including microRNA-21 (miR-21), which was increasingly upregulated from normal liver to MASH to MASH-HCC. We generated transgenic zebrafish that overexpress or sponge miR-21 in hepatocytes. We found that miR-21 overexpression caused larval liver overgrowth and increased HCC, while miR-21 sponge suppressed β-catenin-driven larval liver overgrowth. By performing histological and lipidomics analysis, we found that overexpression of miR-21, like activated β-catenin (ABC), suppressed lipid accumulation in response to a high cholesterol diet and increased accumulation of acylcarnitines. Thus, miR-21, which is similarly upregulated in human and zebrafish HCC, promotes lipid metabolic changes that may help drive hepatocarcinogenesis.

## Teaser

**Summary:** This article provides insights into mechanisms of hepatocellular carcinoma. Our findings suggest that microRNA-21 is a target of activated β-catenin that mediates some of its effects during liver tumorigenesis.

## INTRODUCTION

Hepatocellular carcinoma (HCC) is the third-leading cause of cancer-related death globally and maintains a median 5-year survival of just 16% (Rumgay et al., 2022; Kalasekar et al., 2021). HCC incidence is increasing in parallel with the growing prevalence of obesity and related risk factors, including dyslipidemia, type 2 diabetes and the proinflammatory state that accompanies the metabolic syndrome. These metabolic dysregulations are associated with the development of metabolic dysfunction-associated steatotic liver disease (MASLD) (Kokkorakis et al., 2023), previously referred to as non-alcoholic fatty liver disease (NAFLD) (Kanwal et al., 2024). Advanced stages of MASLD comprise metabolic dysfunction-associated steatohepatitis (MASH), previously known as non-alcoholic steatohepatitis (NASH). MASH is characterized by hepatic inflammation, fibrosis and the accumulation of lipotoxic lipid species, such as acylcarnitines (Melone et al., 2018), ceramides (Chaurasia et al., 2019) and unesterified cholesterol (Beltroy et al., 2005), culminating in cirrhosis. MASH cirrhosis is associated with an annual cumulative HCC incidence of 2.6%, i.e. MASH-driven HCC (hereafter referred to as MASH-HCC) (Ascha et al., 2010). The molecular mechanisms driving the transition from MASH to MASH-HCC are incompletely understood (Wang et al., 2025).

Mutations leading to the stabilization of the Wnt pathway co-transcriptional activator β-catenin (CTNNB1) are among the most frequent oncogenic events in MASH-HCC, occurring in ∼30% of tumors (Cancer Genome Atlas Research Network, 2017; Nault et al., 2020). Activated CTNNB1 [hereafter referred to as activated β-catenin (ABC)] increases fatty acid oxidation and glutamate metabolism (Senni et al., 2019; Cadoret et al., 2002; Monga, 2011). Transgenic zebrafish expressing hepatocyte-specific ABC develop HCC with ∼80% penetrance as adults; zebrafish ABC-HCC is morphologically, transcriptomically and metabolically similar to human HCC (Evason et al., 2015; Kalasekar et al., 2019). ABC zebrafish show robust liver enlargement by 6 days of age due to hepatocyte hyperproliferation, providing a facile platform for testing the effects of drugs and genetic manipulations on an HCC-related phenotype (Evason et al., 2015; Helal et al., 2021).

We have previously reported that ABC causes significant changes to acylcarnitines and triglycerides in cultured human liver cancer cells and in zebrafish liver (VanSant-Webb et al., 2024). ABC also promotes the oxidation of triglycerides and regulation of PPARα (also known as NR1C1 and PPARA) signaling in mice (Senni et al., 2019). Pharmacological inhibition of fatty acid oxidation (FAO) in mice reduces HCC growth (Senni et al., 2019). Results obtained from lipidomics using samples from patients with HCC suggest that there is an increase in fatty acid oxidation during the transition from MASH to MASH-HCC (Rodrigues et al., 2023). Together these studies support the hypothesis that ABC promotes fatty acid oxidation to drive HCC; however, the mechanism(s) underlying the effect of ABC on fatty acid oxidation and other aspects of lipid metabolism are not well defined.

Mature microRNAs (miRNAs) are 20- to 22-nucleotide-long RNA molecules, which are processed from precursor miRNAs (pre-miRNAs) and regulate the transcriptome (Ardekani and Naeini, 2010) by promoting mRNA degradation through direct interaction of conserved sequences between the miRNA and the 3′ UTR of mRNAs. Each miRNA can target numerous genes (Komatsu et al., 2023), and both direct and downstream effects of miRNAs can cause changes to gene expression (O'Brien et al., 2018; Catalanotto et al., 2016). Several miRNAs – including miR-21 (Callegari et al., 2015; Qu et al., 2019), miR-122 (Thakral and Ghoshal, 2015), miR-33 (Fernández-Tussy et al., 2024) and others (Koustas et al., 2023) – were shown to be dysregulated in serum and/or liver tissue of MASH-HCC or HCC patients. The liver readily takes up oligonucleotides, facilitating hepatic delivery of miRNA mimics and antagonists, such as miR-122 inhibitors, which safely and effectively reduce hepatitis C virus infection in the clinic (Thakral and Ghoshal, 2015; Janssen et al., 2013). Thus, miRNAs are enticing targets for HCC therapy.

Here we confirmed that miR-21 expression is increasingly dysregulated from normal liver to MASH and to MASH-HCC in patient tissues, providing further evidence towards the role of miR-21 in MASH-HCC. We discovered that miR-21 is also upregulated in zebrafish HCC driven by ABC (ABC-HCC). We showed that overexpression (OE) of hepatocyte-specific miR-21 (miR-21OE) enhances ABC-driven larval liver enlargement and promotes HCC in adult zebrafish. We found that miR-21OE and ABC trigger similar decreases in hepatic steatosis in response to high-cholesterol diet (HCD). We also revealed that miR-21OE drives HCC-associated lipid metabolic changes, providing insights into mechanisms of MASH-HCC.

## RESULTS

### Identifying conserved dysregulated miRNAs in human and zebrafish HCC

To identify dysregulated, therapeutically relevant miRNAs in MASH and MASH-HCC, we utilized Nanostring to analyze patient liver samples from the University of Utah Pathology Archives (*n*=4 or *n*=7 for each group) (Table S1). Out of the 827 mature miRNAs analyzed with Nanostring/nSolver, levels of 28 miRNAs (14 up, 14 down) were significantly altered in MASH cirrhotic compared to non-cirrhotic control samples, and levels of 31 miRNAs (20 up, 11 down) were significantly altered in MASH-HCC compared to adjacent non-tumor patient samples (Table S2 Workbook S2a). Four miRNAs – let-7f, miR-15b, miR-21 and miR-32 – were significantly upregulated in both MASH and MASH-HCC, suggesting progressive upregulation with advancing liver disease (Table 1, Table S2 Workbook S2a).

**Table 1.** miR-21 levels in MASH, MASH-HCC, TCGA HCC and transgenic zebrafish models of HCC

Tissue	L2FC
MASH versus healthy control	1.80*
MASH-HCC versus adjacent non tumor	1.43*
TCGA HCC versus adjacent non tumor	1.39^+^
ABC versus WT	2.03^+^

ABC, hepatocellular carcinoma in zebrafish expressing hepatocyte-specific activated β-catenin; L2FC, log2 fold-change; MASH, metabolic dysfunction-associated steatohepatitis; MASH-HCC, MASH-driven hepatocellular carcinoma; TCGA HCC, The Cancer Genome Atlas hepatocellular carcinoma; WT, normal liver in wild-type zebrafish; *, false discovery rate (FDR) <0.05; ^+^, *P*<0.0001.

We next validated our findings utilizing miRNA data from The Cancer Genome Atlas Liver and Hepatocellular Carcinoma (TCGA- LIHC) database (Cancer Genome Atlas Research Network, 2017). We removed any patient samples that did not have paired sequencing, samples from patients with a diagnosis other than or in addition to HCC, or samples from patients who had received prior treatment, leaving 45 patient samples for analysis (Table S2 Workbook S2b). Out of 623 miRNAs, levels of 303 (184 up, 119 down) were significantly dysregulated, including those of miR-21 (Table 1, Table S2 Workbook S2c).

To identify miRNAs with a conserved role in hepatocarcinogenesis across species and to focus on those that might mediate the effects of ABC, we profiled miRNAs in zebrafish ABC-HCC. We performed pre-miRNA sequencing and DESeq analysis (Love et al., 2014) of 4-month-old male ABC-HCC zebrafish and their wild-type non-transgenic control siblings (WT) (*n*=5 per group). Out of 212 detected pre-miRNAs, levels of 82 (43 up, 39 down) were significantly dysregulated, including dre-miR-21-1 [log2 fold-change (L2FC) 2.0, adjusted *P*-value (padj)=1.5e-14] and dre-miR-21-2 (L2FC 1.8, padj=8.7e-7) (Table 1, Table S2 Workbook S2d). We used miRbase (Kozomara et al., 2019) and identified 104 homologous miRNAs present in the 827 human miRNAs analyzed with Nanostring and in the 212 zebrafish pre-miRNAs identified by miR-seq (Table S2 Workbook S2e). Fisher's product method was used to compare evidence from human MASH-HCC and zebrafish ABC-HCC differential expression analyses. Of the 104 homologous miRNAs, we identified 26 – including miR-21 – that had concordant differential expression in both humans and zebrafish (Table S2 Workbook S2f). Together, our analyses confirmed that miR-21 is one of the most robustly upregulated miRNAs in MASH-HCC, suggesting shared mechanisms of miRNA-based modulation of HCC in zebrafish and humans.

### Modeling dysregulated miRNAs in zebrafish HCC

**Fig. 1.**
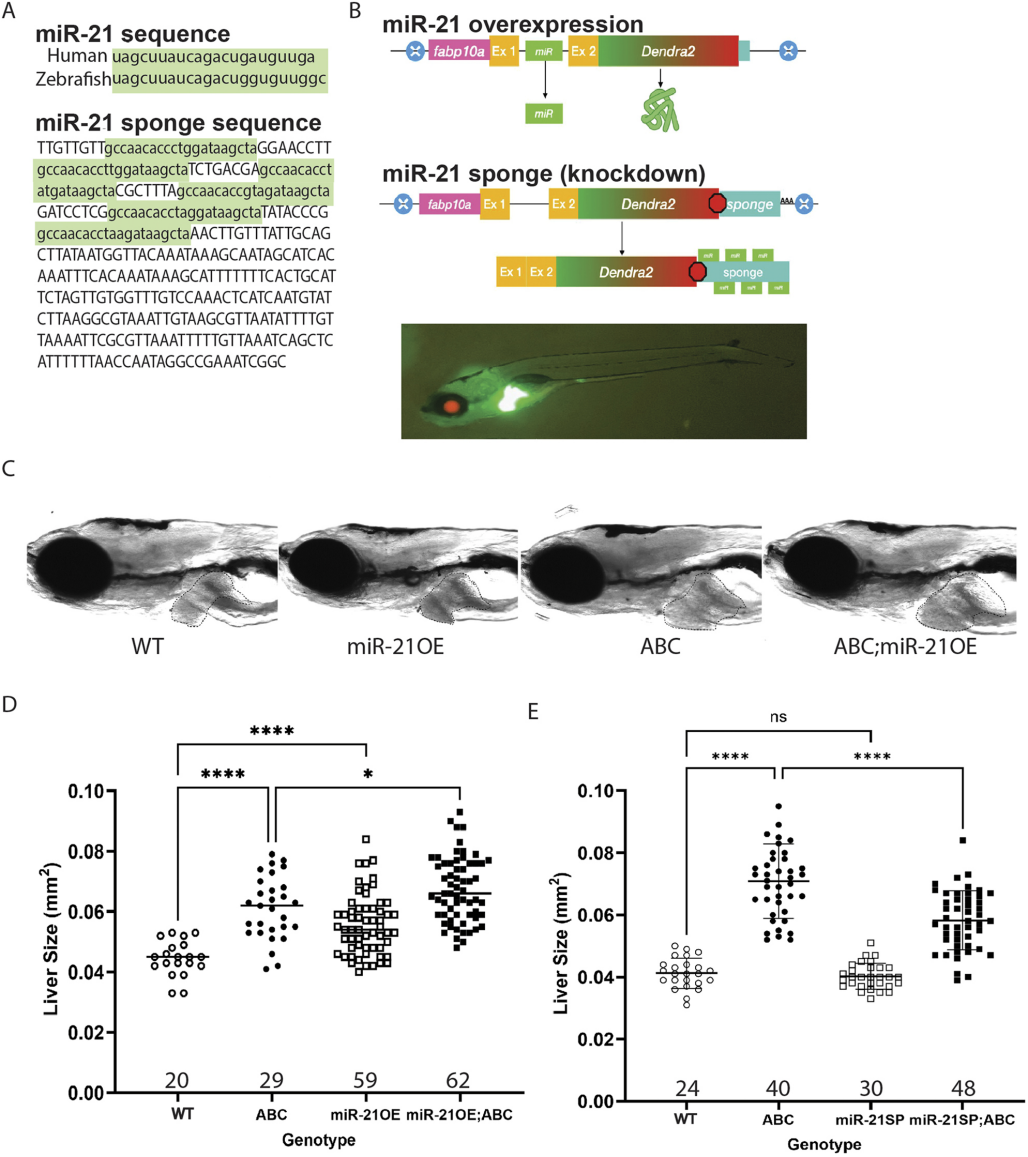
**miR-21 overexpression or sponge alters zebrafish larval liver size compared to non-transgenic wild-type siblings (WT).** (A) Comparison of the human miR-21 (hsa-mir-21-5p) sequence to that from zebrafish (dre-mir-21-5p) and specific sequences used for sponging miR-21. (B) Schematic of plasmid constructs and image of a zebrafish larva with fluorescent red eyes and green liver indicating transgene expression. (C) Representative brightfield images depicting larval zebrafish as indicated at 6 dpf; livers are outlined in black. (D,E) Plots of liver sizes at 6 dpf comparing liver size of miR-21-overexpressing zebrafish (miR-21OE) (D) and miR-21 sponge zebrafish (miR-21SP) (E) to that of WT zebrafish in the presence and absence of ABC. *n* values are shown above the *x*-axes. Representative experimental data are shown. At least three replicates of experiments shown in D and E were performed, showing similar results. *P*-values were determined using GraphPad Prism, Brown-Forsythe and Welch ANOVA, Dunnett's T3 multiple comparisons test: ns, not significant; *P<0.05; ****P<0.001.

To characterize the role of miR-21 in zebrafish hepatocarcinogenesis, we generated transgenic zebrafish lines in which miR-21 is either overexpressed (miR-21OE), i.e. *Tg(fabp10a:Dendra2miR-21OE;cryaa:mCherry)* or ‘sponged’ (SP) (miR-21SP), i.e. *Tg(fabp10a:Dendra2miR-21SP;cryaa:mCherry)*, with miR-21 being under the control of the hepatocyte-specific *fabp10a* promoter (Denovan-Wright et al., 2000) (Fig. 1A,B). We found that miR-21OE significantly increased larval liver size at 6 days post fertilization (dpf) from 0.045 mm^2^ to 0.056 mm^2^ (24% increase compared to liver size in WT, *P*<0.001) (Fig. 1C,D), while miR-21SP did not alter larval liver size compared to that in WT (Fig. 1E). Moreover, miR-21OE enhanced ABC-driven larval liver overgrowth from 0.061 mm^2^ to 0.067 mm^2^ (10% increase, *P*<0.05) (Fig. 1D). By contrast, miR-21SP attenuated ABC-driven larval liver enlargement from 0.071 to 0.058 mm^2^ (18% decrease, *P*<0.0001) (Fig. 1E).

Histological analysis of zebrafish livers at 12 months of age revealed that, in response to miR-21OE, 8% of had HCC and 62% had mild changes, such as the presence of mild cytological or architectural abnormalities, insufficient for diagnosis of HCC (Evason et al., 2015) (*P*<0.05 compared to WT) (Fig. S1). miR-21OE also led to HCC in adult zebrafish carrying a loss-of-function mutation in the tumor suppressor gene *p53* (*tp53*^M214K^) (Fig. S1) (Berghmans et al., 2005). These results support the hypothesis that miR-21 promotes HCC in zebrafish.

To identify mechanisms by which miR-21 might increase larval liver size, we examined nuclear features including nuclear size, nuclear density and inter-nuclear distance at 6 dpf in miR-21OE and WT control zebrafish by using QuPath (Bankhead et al., 2017). We found that miR-21OE decreased nuclear density from 15.7 nuclei/1000 µm^2^ to 13.6 nuclei/1000 µm^2^ (*P*<0.05) (Fig. S2). By contrast, miR-21OE increased the average inter-nuclear distance from 8.0 µm to 8.6 µm (*P*<0.05) (Fig. S2). There was no significant difference in the size of nuclei (Fig. S2). We did not detect significant differences in DNA synthesis by 5-ethynyl-2′-deoxyuridine (EdU) labeling in miR-21OE zebrafish at 6 dpf (Fig. S3).

We found that miR-21OE and miR-21SP did not impact liver-to-body ratios, liver size or body mass at 10 weeks of age, and miR-21OE did not affect survival (Figs S4 and S5).

### miR-21OE, like ABC, suppresses hepatic lipid deposition in response to HCD in larvae

**Fig. 2.**
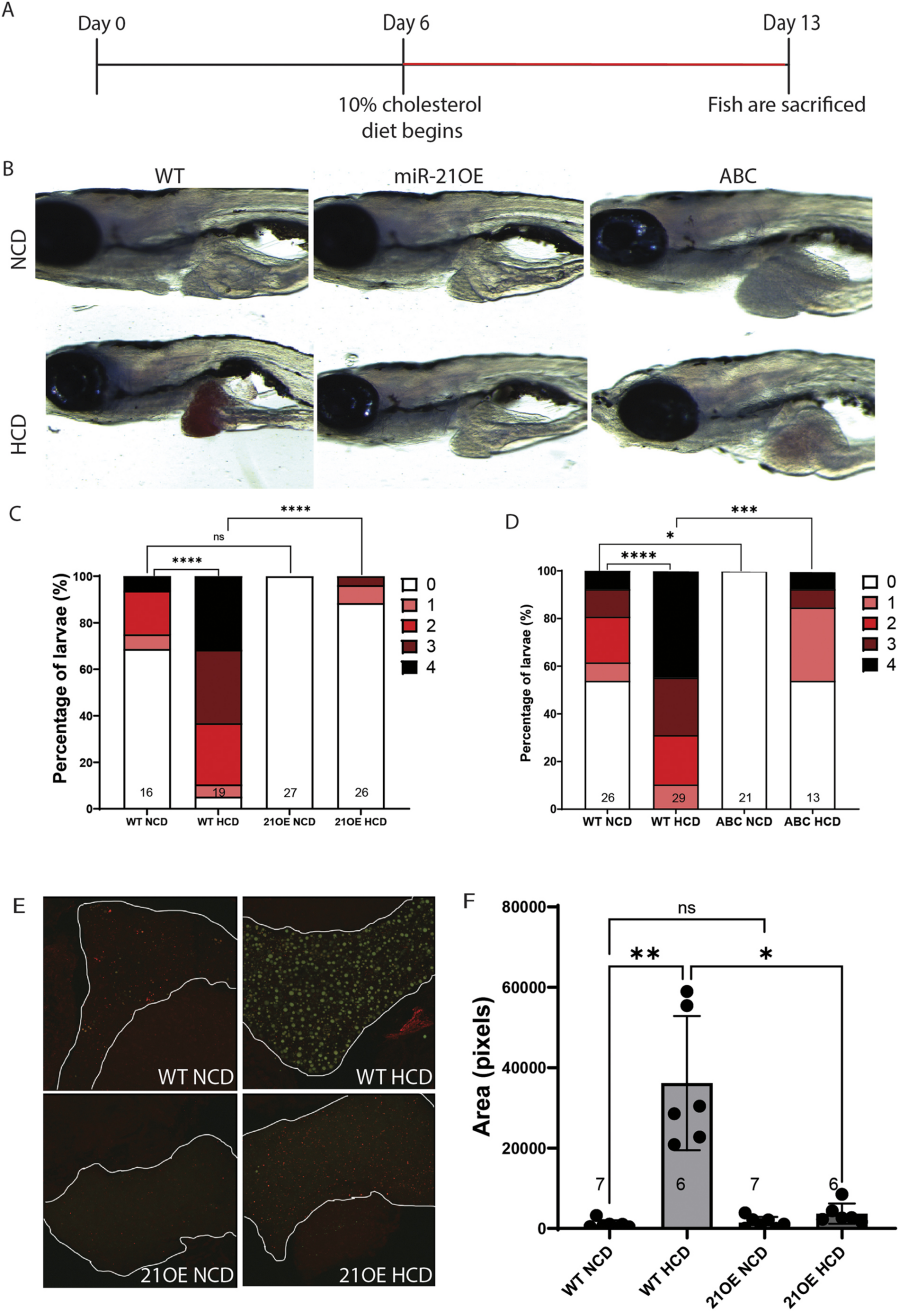
**miR-21OE decreases steatosis in larval zebrafish fed a high cholesterol diet.** (A) Timeline of diet treatment. (B) Representative brightfield images of 13 dpf miR-21 overexpression (21OE), ABC and non-transgenic wild-type control (WT) sibling zebrafish after Oil Red O staining (ORO). HCD, high cholesterol diet; NCD, normal control diet. (C,D) Quantification of ORO of larval liver images. Scoring is from 0-4, with 4 indicating most intense staining; *n* values are indicated at the *x*-axis at the bottom of each column. *P*-values determined with GraphPad Prism, Kruskal–Wallis test with Dunn's multiple comparisons test: ns; not significant, *P<0.05, ***P<0.001, ****P<0.0001. (E,F) Representative confocal images (E) and quantification of Nile Red stained areas indicating steatosis (F). Livers are outlined in white. *P*-values determined using GraphPad Prism, Brown-Forsythe and Welch ANOVA with Dunnett's T3 multiple comparisons test: ns, not significant; ****P<0.0001. We performed three (C), two (D) or three (E,F) replicates of each experiment, and data from one representative experiment are shown.

To understand the role of miR-21 in hepatic lipid metabolism in zebrafish, including under conditions of metabolic stress, we fed miR-21OE larval zebrafish and WT siblings a normal control diet (NCD) comprising GEMMA Micro 75 zebrafish food or a HCD created by supplementing this commercial diet with 10% by weight cholesterol (De Oliveira et al., 2019) (Fig. 2A). We quantified steatosis by masked examination of larval sections stained with Hematoxylin and Eosin (H&E), as well as whole-mount staining of larvae with Oil Red O (ORO) and Nile Red [as described by Ramírez-Zacarías et al. (1992) and Khan et al. (2024)]. We also analyzed sections of H&E-stained larvae for inflammatory clusters to assess inflammation.

ORO staining highlights neutral lipid droplets that predominantly comprise triglycerides and cholesterol oleate (Ramírez-Zacarías et al., 1992), and has not been described to stain lipotoxic lipid species, such as acylcarnitines, ceramides and unesterified cholesterol. Nile Red staining is more sensitive than ORO staining, and can detect lipid and phospholipid droplets on the basis of excitation and emission settings used for imaging (Khan et al., 2024). In WT zebrafish, HCD increased steatosis as assessed by staining with ORO (*P*<0.0001), Nile Red (*P*<0.0001) and H&E (*P*<0.0001) (Fig. 2B-F, Figs S6, S7). HCD also increased inflammation in WT zebrafish as indicated by inflammatory clusters (*P*<0.0001) (Fig. S7b). miR-21OE suppressed HCD-induced steatosis, indicated by decreased ORO staining (*P*<0.0001), decreased Nile Red staining (*P*<0.0001) and H&E (*P*<0.001) (Fig. 2B-F, Figs S6, S7). ABC-HCC zebrafish on HCD also showed decreased ORO staining compared to WT fish on HCD (*P*<0.0001) (Fig. 2B,D). At 13 dpf, miR-21OE did not affect HCD-induced inflammation (Fig. S7) and miR-21SP did not significantly affect HCD-induced steatosis or inflammation (Fig. S8). Overall, miR-21OE suppressed fat droplet accumulation in zebrafish liver in a manner analogous to ABC, suggesting common effects on lipid metabolism by both.

### HCD alters lipid metabolism genes in larval zebrafish

**Fig. 3.**
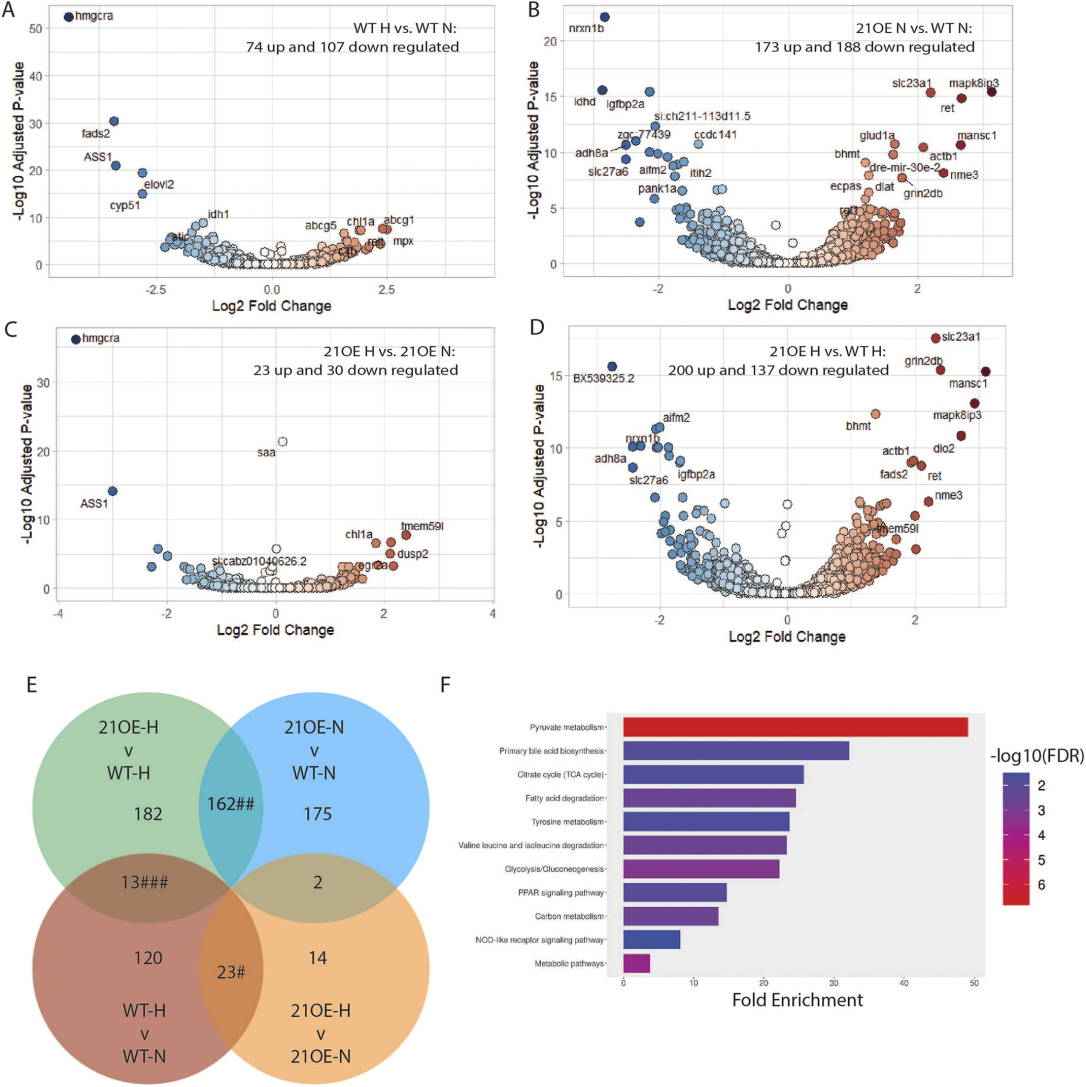
**Gene expression changes induced by miR-21OE and/or high-cholesterol diet in larval zebrafish.** (A-D) Volcano plots generated from RNA-seq data of livers obtained at 13 dpf from miR-21OE (21OE) and/or non-transgenic wild-type control sibling (WT) zebrafish larvae administered a normal control diet (N) or high-cholesterol diet (H). (E) Select intersection comparison of RNA-seq analysis (Table S3 Workbook S3e). #, diet-dependent genes; ##, miR-21-dependent genes; ###, miR-21- and HCD-dependent genes (Table S3 Workbooks S3f,g,h). (F) Significant KEGG pathways of downregulated miR-21-dependent genes. (*n*=67, FDR<0.05) (Tables S3, S4 Workbooks S3g, S4j).

To identify potential targets of miR-21, including those dependent on diet, we performed RNA sequencing of livers dissected from 13-day-old miR-21OE zebrafish and WT siblings that had been on NCD or HCD (Fig. 3A-D, Table S3). Overall, 181 genes (74 up, 107 down) were significantly dysregulated when comparing WT-HCD versus WT-NCD (Table S3 Workbook S3a). ShinyGO v0.85 (Ge et al., 2020) was utilized to perform species-specific analysis with Ensembl IDs using both Gene Ontology (GO) Biological Process and KEGG Pathway Analysis, for dual verification, both of which identified dysregulated cholesterol synthesis pathways (“Sterol biosynthetic process” GO:0016126, FDR=1.5E-06, 45.4 Fold Enrichment; “Steroid biosynthesis” KEGG dre 00100, FDR=5.2E-16, 82.6 Fold Enrichment) (Table S4 Workbook S4a).

To further investigate gene expression changes induced by HCD in zebrafish, we examined changes across genotype and diet (Fig. 3E, Fig. S9, Table S3). We considered genes to be diet-dependent if they were significantly dysregulated in both WT-HCD versus WT-NCD (Table S3 Workbook S3a) and miR-21OE-HCD versus miR-21OE-NCD (Table S3 Workbook S3d), but not altered by miR-21OE (Table S3 Workbooks S3b,c). In total, 23 genes (9 up, 14 down) were significantly dysregulated in a diet-dependent manner (Fig. 3E, Table S3 Workbook S3e and S3f). Pathway analysis of the 23 genes identified “Steroid biosynthesis” KEGG dre00100 (FDR 1.8E-5, 178.8 Fold Enrichment) (Table S4 Workbook S4b).

We next examined similarities between larval zebrafish fed a HCD and mice with metabolic dysfunction-associated steatotic liver disease (MASLD). For this analysis, we included RNA-seq data from additional WT-HCD and WT-NCD samples (six of each group), which were combined with our initial data (three samples of each group), analyzed with DESeq, and compared to non-tumorous diet-induced obese (DIO) mice versus chow-fed control mice (GSE230639) (Tsouka et al., 2024) (Table S5). We found 617 genes (304 up, 187 down, 126 discordant) that were dysregulated in both zebrafish and mouse MASLD models (*P*=7E-13 Fisher's exact test) (Fig. S10, Table S5 Workbook S5c).

### miR-21OE dysregulates metabolic genes in larval zebrafish

We next turned our attention to gene expression changes driven by miR-21 overexpression. We found that miR-21OE-NCD zebrafish had significantly higher levels of dre-mir-21-1 compared to WT-NCD controls (L2FC 1.73, padj<0.01), confirming overexpression of miR-21 through the miR-21OE transgene construct (Table S3 Workbook S3b). We identified 361 genes (173 up, 188 down) that were significantly dysregulated when comparing miR-21OE-NCD versus WT-NCD (Table S3 Workbook S3b). To find potential target pathways of miR-21OE, we used ShinyGO on the 188 downregulated genes (Table S4 Workbook S4d). We discovered enrichment of “Carboxylic acid metabolic process” (GO:0019752, FDR=8.0E-04, 5.5 Fold Enrichment), “Carbon metabolism” (KEGG dre01200, FDR=3.1E-7, 12.3 Fold Enrichment), “Pyruvate metabolism” (KEGG dre00620, FDR=7.5E-8, 23.8 Fold Enrichment), and “Glycolysis/Gluconeogenesis” (KEGG dre00010, FDR=1.7E-4, 12.1 Fold Enrichment) among other metabolic pathways. There was also enrichment for “PPAR signaling pathway” (KEGG dre03320, FDR=3.0E-5, 12.5 Fold Enrichment).

When comparing miR-21OE-HCD versus WT-HCD zebrafish larvae, 337 genes (200 up, 137 down) were significantly dysregulated (Table S3 Workbook S3c). ShinyGO pathway analysis of the 137 downregulated genes found enrichment of “Regulation of insulin-like growth factor receptor signaling pathway” (GO:0043567, FDR=5.6E-3, 76.7 Fold Enrichment) and “Pyruvate metabolism” (KEGG dre00620, FDR=1.1E-5, 25.1 Fold Enrichment) (Table S4 Workbook S4g).

Since similar genes and pathways – including those involved in pyruvate metabolism – were impacted in miR-21OE compared to WT zebrafish larvae under both dietary conditions; we examined miR-21OE-induced changes across genotype and diet (Fig. 3E). Genes were considered to be dependent on miR-21OE if they were similarly significantly dysregulated in both miR-21OE-NCD versus WT-NCD, and miR-21OE-HCD versus WT-HCD (Table S3 Workbooks S3b,c). In total, 162 genes (95 up, 67 down) were significantly dysregulated in a miR-21OE-dependent manner (Fig. 3E, Table S3 Workbook S3g). Using TargetScan (Lewis et al., 2005; Ulitsky et al., 2012; Grimson et al., 2007; Friedman et al., 2009), we found that 23 out of 67 (34%) of the downregulated miR-21-dependent genes contained one or more miR-21 seed sequences in their 3′ UTRs (Table S3 Workbook S3i). We confirmed expression of five of these genes in miR-21OE-NCD and WT-NCD by qRT-PCR in independent experiments (Fig. S11). ShinyGO analysis of the 67 downregulated genes showed negative enrichment for “PPAR signaling pathway” (KEGG dre03320, FDR=9.5E-3, 14.7 Fold Enrichment) and “Pyruvate metabolism” (dre00620, FDR=1.5E-7, 49.1 fold enrichment) (Fig. S12 and Table S4 Workbook S4j).

To define how effects of miR-21OE might diverge from a physiologic response to HCD, we performed ShinyGO analysis of the 17 genes that were significantly dysregulated in opposite directions in WT-HCD versus WT-NCD, and miR-21OE-HCD versus WT-HCD, zebrafish larvae (Table S3 Workbooks S3a,c). Four of these genes were also dysregulated in miR-21OE-HCD versus miR-21OE-NCD fish (Table S3 Workbook S3d). We found enrichment of “Biosynthesis of unsaturated fatty acids” (KEGG dre01040, FDR=1.8E-3, 110.9 Fold Enrichment) due to dysregulation of *fads2* and *elovl2* (Table S4 Workbook S4l). Gene set enrichment analysis (GSEA) with C3 TFT motif analysis (20% false discovery rate) did not identify a gene signature or negative gene enrichment pattern consistent with miR-21 degradation of specific transcription factors (Table S6) (Korotkevich et al., 2021 preprint; https://bioconductor.org/packages/release/bioc/html/fgsea.html).

### miR-21OE and ABC dysregulate similar genes in adult zebrafish, including *pparab*

The above analyses identified downregulation of zebrafish *PPARA* orthologs *pparaa* (WT-HCD versus WT-NCD) and *pparab* (miR-21OE-NCD versus WT-NCD), as well as negative enrichment of “PPAR signaling pathway” (KEGG analysis of the 67 genes that were downregulated in a miR-21OE-dependent manner) (Tables S3, S4). The finding that *pparaa* and *pparab* were downregulated in response to both HCD and miR-21OE, respectively, prompted us to test the hypothesis that pharmacological targeting of *pparaa* and/or *pparab* can rescue phenotypes associated with miR-21 dysregulation. We found that the PPARα agonist bezafibrate decreased miR-21OE-driven larval liver overgrowth from 0.031 mm^2^ to 0.025 mm^2^ (19% decrease, *P*<0.0001) (Fig. S14).

We have previously reported that *pparab* is also significantly downregulated in adult male ABC-HCC zebrafish (Kalasekar et al., 2019), encouraging us to investigate other similarities between miR-21OE and ABC. For this, we compared the significantly dysregulated genes in the liver from miR-21OE-NCD and WT-NCD (Table S3 Workbook S3b) to that from 6-month-old adult male ABC-HCC versus WT control zebrafish (Kalasekar et al., 2019), and from 4- to 6-month-old adult female ABC-HCC versus WT control zebrafish (Evason et al., 2015) by using SuperExactTest v1.1 (Wang et al., 2015). We found overlap between each dataset pairing, as well as between the multi-set interaction of the three datasets, showing an overlap of 26 genes (Fold enrichment 2.05, *P*=4.70e-4) (Fig. S15, Table S7 Workbook S7).

### miR-21OE, like ABC, suppresses hepatic lipid deposition in response to HCD in adults

**Fig. 4.**
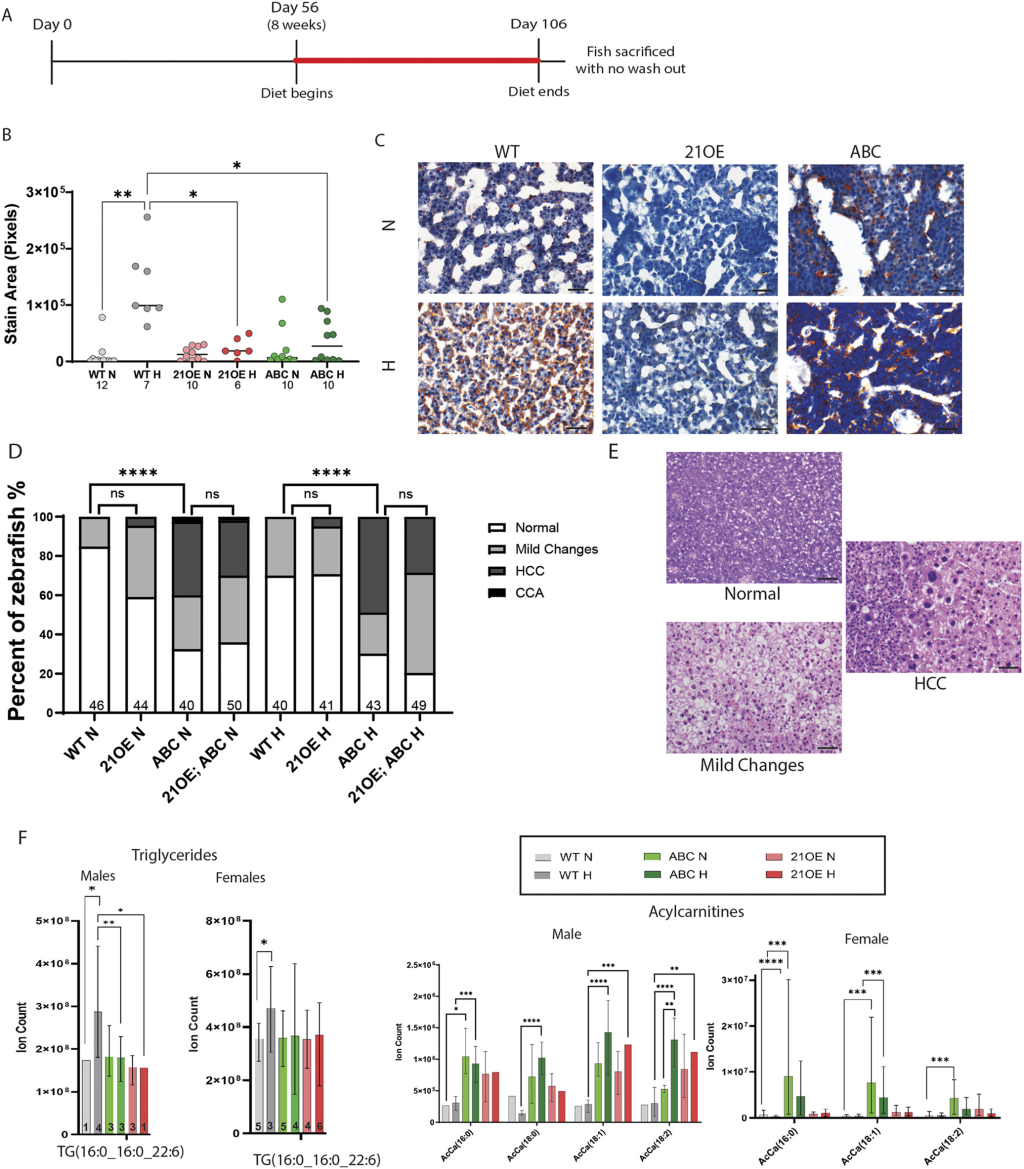
**miR-21OE and ABC decrease steatosis, decrease triglycerides, and increase acylcarnitines in adult zebrafish on a high cholesterol diet.** (A) Timeline for feeding high cholesterol diet (H) or normal control diet (N) to adult zebrafish. (B) Quantification of ORO for miR-21 overexpression (21OE), ABC, and non-transgenic wild-type control (WT) sibling zebrafish livers. *n* values are indicated below the *x*-axis for each column. *P*-values determined with GraphPad Prism, Brown-Forsythe and Welch ANOVA with Dunnett's T3 multiple comparisons test: *P<0.05; **P<0.01. (C) Representative ORO images. (D,E) Liver histology was assessed by Hematoxylin and Eosin (H&E) staining, with representative images shown in E. Data quantification is shown in D; *n* values are indicated within each column above the *x*-axis. *P*-values were determined with GraphPad Prism, Kruskal–Wallis test with Dunn's multiple comparisons test (ns, not significant; ****P<0.0001). (F) Plotted are the most-abundant triglyceride (left) and most-abundant acylcarnitine counts (right), with *n* values indicated within each column above the *x*-axis in triglyceride counts plots (left). *P*-values were determined by using GraphPad Prism (two-way ANOVA, *P<0.05, **P<0.01, ***P<0.001, ****P<0.0001). All other comparisons were statistically not significant. Three independent replicates were performed for these experiments. One representative experiment has been plotted in graph B. Pooled experimental data from three experiments are plotted in D and F. Complete triglyceride and acylcarnitine data shown in Fig. S20. Scale bars: 20 μm.

To further define the effects of miR-21OE and ABC on lipid metabolism, we administered 10% HCD or NCD to adult zebrafish (Fig. 4A). In WT zebrafish, HCD increased hepatic lipid deposition (as quantified by ORO staining; *P*<0.01) but did not cause HCC (Fig. 4B-E, Figs S16, S17). Similar to larval zebrafish, adult ABC-HCD and miR-21OE-HCD showed reduced lipid deposition compared to WT-HCD zebrafish (*P*<0.05 and *P*<0.05, Fig. 4B,C), and miR-21SP-HCD did not significantly alter lipid deposition (Fig. S18). ABC-NCD male and female zebrafish had more-severe histologic abnormalities, including higher HCC incidence (83% and 18%, respectively) compared to male and female WT-NCD zebrafish (both 0%, *P*<0.001) (Fig. 4D, Fig. S17), confirming our previously published observations (Evason et al., 2015; Kalasekar et al., 2019). HCC incidence was not significantly altered by HCD in any genotype or sex (Fig. S17). miR-21OE zebrafish rarely showed HCC and HCC was not observed in WT, with this difference not being statistically significant (Fig. 4D, Fig. S17), and miR-21OE did not significantly alter HCC incidence in the presence of ABC (Fig. 4D, Fig. S17). These data demonstrated that miR-21OE, like oncogenic ABC, suppresses normal lipid droplet accumulation in response to HCD.

### miR-21OE, like ABC, leads to decreased levels of triglycerides and increased levels of acylcarnitines

We next analyzed changes to the lipidome in adult miR-21OE, ABC and WT zebrafish to which NCD or HCD had been administered (Fig. S19). The most striking diet-induced changes in WT-HCD were in triglycerides (TGs), with the most-abundant TG (16:0_16:0_22:6), increased in both males (*P*<0.05) and females (*P*<0.05) (Fig. 4F, Fig. S19). Both ABC and miR-21OE suppressed the HCD-induced increase in TGs, although this effect was only statistically significant in males (Fig. 4F, Fig. S19).

In ABC fish, the largest changes were to acylcarnitines (AcCa), regardless of diet. Male and female ABC zebrafish on either NCD or HCD showed significant increases in AcCa (16:0) [males: *P*<0.05 (NCD), *P*<0.001 (HCD); females: *P*<0.0001 (NCD), *P*<0.001 (HCD)] (Fig. 4F). Other abundant AcCa species, including AcCa (18:0), AcCa (18:1) and AcCa (18:2) were also significantly increased in ABC zebrafish on HCD. miR-21OE zebrafish also tended to show increased AcCa compared to WT controls, although this effect was only statistically significant for AcCa (18:1) (*P*<0.0001) and AcCa (18:2) (*P*<0.001) in male zebrafish on HCD (Fig. 4F, Fig. S19).

## DISCUSSION

Our work adds to the growing literature reporting that miRNAs are dysregulated in context-dependent states that change over time as liver disease progresses, and we highlight the utility of zebrafish to model miRNA dysregulation. Specifically, here we showed that miR-21 is upregulated in liver tissue from patients with MASH and MASH-HCC, as well as in zebrafish ABC-HCC, supporting a conserved role for miR-21 in hepatocarcinogenesis. Our findings in MASH patients confirmed the findings by Rodrigues et al., who found that miR-21 levels increased in a European cohort of patients with MASLD and MASH (Rodrigues et al., 2023). Our finding that miR-21 is also upregulated in MASH-HCC compared to adjacent non-tumor tissue, together with prior results that have indicated a positive correlation between miR-21 levels and age (Rodrigues et al., 2023), suggests progressive dysregulation of miR-21 during MASH-driven hepatocarcinogenesis.

We found that miR-21 overexpression was sufficient to promote zebrafish larval liver overgrowth and enhance ABC-driven larval liver enlargement. miR-21 overexpression increased HCC in adult zebrafish, albeit to a lesser extent than ABC. Sponging miR-21 suppressed ABC-driven larval liver enlargement but did not affect larval liver size in the absence of ABC; this finding may be due to relatively low expression of miR-21 in healthy, WT zebrafish liver. We discovered that overexpression of miR-21 in zebrafish liver caused significant changes to genes involved in lipid and glutamate metabolism, which are also dysregulated by ABC (Nault et al., 2020; Senni et al., 2019). In human glioma cells and umbilical vein endothelial cells, ABC regulates miR-21 expression indirectly via STAT3 (Han et al., 2012). ABC has also been shown to directly regulate miR-21 transcription, as the β-catenin co-transcription factor TCF4 binds the miR-21 promoter region in human breast, colon and glioma cells (Lan, 2012). Together, these findings suggest that miR-21 is a direct or indirect downstream target of ABC, thereby mediating some of the effects of ABC during liver tumorigenesis.

ABC increases hepatocyte proliferation in larval zebrafish (Evason et al., 2015). We observed a slight increase in nuclear size and proliferation with miR-21OE, but neither change was statistically significant. The finding of increased inter-nuclear spacing and/or decreased nuclear density in miR-21OE zebrafish supports the hypothesis that miR-21OE larval liver enlargement is due, at least in part, to increased hepatocyte size, potentially related to miR-21OE-driven metabolic changes, i.e. hepatocyte hypertrophy is associated with increased metabolic or synthetic demand, altered lipid handling, metabolic reprogramming, cellular stress, and/or early regenerative/growth signaling (Hall et al., 2012; Maronpot et al., 2010; Greaves, 2007; Target Organ Pathology: A Basic Text, 1998).

To understand the intersection of diet, ABC-HCC and miR-21, we administered a 10% HCD to zebrafish, which has been shown to induce hepatic steatosis, immune infiltration and hepatocyte enlargement (De Oliveira et al., 2019; Progatzky et al., 2014; Dai et al., 2015; Jin et al., 2024). Excess cholesterol induces changes to lipid metabolism in the hepatocyte through altered regulation of SREBP2 (Shimano and Sato, 2017; Horton et al., 2002), SREBP1c (Bertolio et al., 2019) and the assortment of PPAR transcription factors (Kersten, 2008). We confirmed histologically that HCD induced hepatic steatosis and inflammation, and increased expression of genes in pathways related to sterol and cholesterol processes (Tables S3, S4). HCD led to downregulation of *ldlra*, *hmgcra* and *fads2* (Tables S3, S5 Workbooks S3a, S5a), which are involved in *de novo* cholesterol synthesis (Brown and Goldstein, 1980), cholesterol homeostasis (Gu and Zhang, 2015) and the regulation of polyunsaturated fatty acids (Ge et al., 2003), respectively. There was also downregulation of *srebf2* and *pparaa* in the livers of 13-dpf WT-HCD larvae compared to WT-NCD control larvae (Table S3 Workbook S3a). HCD decreased *stard4* and increased *apoa4*, which are involved in cholesterol esterification (Rodriguez-Agudo et al., 2008) and triglyceride export (VerHague et al., 2013), respectively, and help mediate cross talk between cholesterol and lipid metabolism through *srebp1c* (MacLeod et al., 2024). We found that WT-HCD zebrafish had a significant overlap in dysregulated genes compared to Gubra-Amylin NASH (GAN) diet-induced obese (DIO) mice, supporting the relevance of our findings to mammals (Fig. S10, Table S5) (Hansen et al., 2023; Gerhard et al., 2018; Suppli et al., 2019; Govaere et al., 2020).

When comparing genes that were significantly altered in the presence of miR-21OE, we found diet-independent effects of miR-21, such as downregulation of genes involved in pyruvate metabolism and PPARα signaling, coupled with the upregulation of genes involved in folate metabolism (Tables S3, S4, Fig. S13). On the other hand, some miR-21OE-induced gene expression changes were dependent on diet. For example, *fads2* and *elovl2* – which are involved in triglyceride and long-chain polyunsaturated fatty acid accumulation, respectively (VerHague et al., 2013; Hayashi et al., 2021; Zhang et al., 2022) – were significantly increased with miR-21OE compared to WT fish on HCD. These two genes were lower in HCD- versus NCD-fed zebrafish for both genotypes. Together, these gene expression changes suggest that miR-21OE may rewire metabolism, such that hepatocytes avoid excessive lipid droplet formation in response to excess cholesterol. This hypothesis is corroborated by our ORO, Nile Red and H&E staining, and lipidomics results, showing that miR-21OE decreased accumulation of lipid droplets and neutral lipids, such as triglycerides, in response to HCD. Multiple lines of evidence suggest that suppression of triglyceride accumulation in lipid droplets represents a pathologic tumor-promoting response. First, we noted a similar suppression of lipid accumulation in response to overexpression of ABC, a well-established hepatic oncogenic protein (Cadoret et al., 2002; Xu et al., 2022). Second, in humans (Powell et al., 1990) and mice (Hansen et al., 2023) with MASH, steatosis decreases during late stages, i.e. when HCC rates are highest. Decreased steatosis during progression from MASH to HCC may reflect alterations in fatty acid metabolism, an emerging hallmark of cancer (Ma et al., 2018).

We found acylcarnitines to be the most substantially altered lipid species in response to ABC and miR-21OE, confirming our prior results in zebrafish and human cell lines (VanSant-Webb et al., 2024). Prior studies in mouse osteoblasts (Frey et al., 2018) and mouse HCC (Senni et al., 2019) have also shown that ABC promotes fatty acid oxidation. Serum levels of long-chain acylcarnitines increase during progression from MASLD to MASH-HCC (Enooku et al., 2019; Caponigro et al., 2023) and are elevated in liver tissue from ABC zebrafish compared to that of controls (VanSant-Webb et al., 2024). Our current findings support a positive correlation between acylcarnitine levels and tumor burden. ABC fish had the highest levels of acylcarnitines and the most HCC, miR-21OE had intermediate levels of acylcarnitines and occasional HCC, and WT had the lowest acylcarnitine levels and no HCC. Together these data suggest that, like ABC, miR-21 promotes fatty acid oxidation, potentially resulting in a lipotoxic state that can drive further HCC progression (Nakagawa et al., 2018) and support tumor burden.

Across many cancer types, miR-21 is one of the best-established oncogenic miRNAs (Medina et al., 2010; Rhim et al., 2022; Volinia et al., 2006) Nonetheless, prior HCC studies have yielded seemingly conflicting results regarding whether miR-21 is pro- or anti-tumorigenic in the liver, or if the role of miR-21 changes in time as disease progresses. On one hand, this work and work by others have shown that hepatic miR-21 levels are elevated in MASH-HCC (Rodrigues et al., 2023), and miR-21 knockout decreases liver tumorigenesis in mice fed a choline-deficient diet (Rodrigues et al., 2023). On the other hand, in some situations, miR-21 appears to be protective against HCC: When high-fat diet is combined with the carcinogen diethylnitrosamine (DEN) given at 3 weeks, constitutive whole-body knockdown of miR-21 leads to increased liver tumor burden, while miR-21 mimic decreases liver tumors (Jagtap et al., 2025). Whole-body or hepatocyte-specific miR-21 knockout leads to increased tumorigenesis in response to carcinogen (DEN) exposure or in liver-specific PTEN knockout mice (Correia De Sousa et al., 2021).

This study examined the effects of miR-21OE at various time points and under different dietary conditions, thereby providing some insights into this apparent discrepancy. We propose that in the setting of a non-neoplastic liver – such as with mice fed a choline-deficient diet, adult zebrafish that are not overexpressing another oncogene (Fig. 4, miR-21OE versus WT groups; Fig. S1) or larval zebrafish with β-catenin-driven hepatocyte hyperproliferation (Fig. 1) – miR-21 overexpression drives lipid dysregulation and HCC. However, once HCC has been established or induced via genetic alterations and/or carcinogen treatment (Jagtap et al., 2025; Correia De Sousa et al., 2021), miR-21-driven metabolic dysregulation decreases tumor burden by promoting tumor cell death. This hypothesis is supported by prior reports that miR-21 knockout decreases TUNEL staining and active caspase-2 levels in mice that were fed a methionine-choline deficient diet (Rodrigues et al., 2017). Future work in our laboratory will focus on defining the link between miR-21, metabolism, cell death and liver tumorigenesis.

## MATERIALS AND METHODS

### Statement of ethics

Zebrafish (*Danio rerio*) studies were performed in compliance with the University of Utah Institutional Animal Care and Use Committee guidelines (Protocols #1809 and #2233) under the supervision of Office of Comparative Medicine veterinarians and staff. Studies on human tissues were reviewed and deemed exempt by the University of Utah Institutional Review Board (IRB #00091019).

### miRNA expression analysis in University of Utah patient samples

We searched the University of Utah Pathology Archives and identified samples from seven patients with MASH-HCC, four patients with MASH (and no clinical or pathological evidence of HCC) and four control patients without cirrhosis or MASH (Table S1). Patients were excluded if they had a history of infection with hepatis B or C virus, or significant alcohol use. The University of Utah High-Throughput Genomics (HTG) Shared Resource Core extracted RNA from paraffin-embedded, formalin-fixed liver tissues using the Qiagen miRNeasy FFPE kit and assessed miRNAs using the Nanostring nCounter Human v3 miRNA Expression Assay kit (CSO-MIR3-12). miRNA counts were analyzed using nSolver v4.0.70 with a filter of 20 reads. miRNAs were considered significantly dysregulated with log2 fold-change (L2FC)±0.5 and FDR<0.05 (Table S2 Workbook S2a).

### miRNA expression analysis in The Cancer Genome Atlas patient samples

To determine significantly dysregulated miRNAs in patients with HCC, human paired tumor and non-tumor miRNA expression quantification normalized count data was gathered from The Cancer Genome Atlas Liver Hepatocellular Carcinoma (TCGA-LIHC) GDC 23.0 Data Release using TCGA biolinks package (Colaprico et al., 2016). Patient samples were excluded from analysis if history indicated that they had received treatment prior to biopsy or had a final diagnosis of a malignancy other than, or in addition to, HCC. In total, 45 patients were eligible for analysis (Table S2 Workbook S2b). Differentially expressed miRNAs were identified with DESeq2 version 1.34.0 (Love et al., 2014). miRNAs were considered significantly dysregulated with a L2FC±0.5 and padj<0.05 (Table S2 Workbook S2c).

### Zebrafish husbandry

Zebrafish (*D. rerio*) lines were maintained under standard conditions (Kimmel et al., 1995). In brief, embryos and larvae were cultured in egg water (2.33 g Instant Ocean in 1 l Milli-Q water with 0.5 ml Methylene Blue) or low-salt egg water (60 mg Instant Ocean in 1 l Milli-Q water) and incubated at 28.5°C. At ∼5 days post fertilization (dpf), zebrafish were transitioned to a recirculating system. Juvenile and adult zebrafish were fed brine shrimp, commercial powdered food, GEMMA and/or commercial powdered food, and housed on a recirculating system. In addition to wild-type AB zebrafish (WT), we also used the previously established *Tg(fabp10a:pt-β-cat)* zebrafish, which express hepatocyte-specific activated β-catenin (ABC) (Evason et al., 2015).

### miRNA expression analysis in zebrafish livers

Five transgenic male ABC zebrafish and five non-transgenic male wild-type control siblings (WT) were raised under standard conditions to adulthood as described above and euthanized at 4 months of age. Livers were dissected from the body cavity, a small portion of each liver was fixed in 4% PFA and submitted to ARUP Research Histology to generate H&E-stained slides, and the remaining liver was snap frozen. H&E-stained slides were examined by a pathologist (K.J.E.) to confirm the diagnosis of HCC (ABC) or no significant pathologic abnormalities (WT). RNA extraction was done with Direct-zol (Zymo Research). The HTG Shared Resource Core prepared libraries by using the NEBNext Multiplex Small RNA library Prep Set and performed gene sequencing using the Illumina HiSeq 50 bp single-read sequencing system. miRNAs were aligned to GRCz10 and counted by using miRbase. Differentially expressed miRNAs were identified with DESeq2 version 1.34.0 (Thermes et al., 2002). miRNAs were considered significantly dysregulated at L2FC ±0.5 and padj<0.05 (Table S2 Workbook S2d).

### Human-to-zebrafish miRNA comparison

Fisher's product method was used to combine evidence from human MASH-HCC and zebrafish ABC-HCC differential expression analyses. Briefly, a χ^2^-squared test statistic was calculated with the following equation:

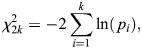
where *k*=2 for two tests.

To adjust for multiple comparison, Benjamini and Hochberg procedure for controlling the false discovery rate (FDR) of a family of hypothesis tests was performed. miRNA was determined to be statically significant if (1) padj≤0.05, and (2) the L2FC for both human and zebrafish were both concordant in differential expression. Matlab v 2023b was used for analyses (MathWorks).

### Generation of transgenic zebrafish to overexpress or sponge miR-21 (miR-21OE and miR-21SP)

To generate the *fabp10a:miR-21* plasmid (*fabp10a-miR-21-1-Dendra2,cryaa:mCherry*), we first made *miR-21-1-BbsI* by amplifying *dre-mir-21-1* from AB zebrafish genomic DNA using primers with BbsI restriction sites (Table S8) and inserting it into *Tol2-lyzC-Vector-Dendra2* (Addgene plasmid #97101, deposited by Qing Deng) (Hsu et al., 2019). From this *Tol2-lyzC-miR-21-1-Dendra2* plasmid, we amplified *miR-21-1-Dendra2* with flanking XhoI restriction sites and placed it downstream of the *fabp10a* promoter (Denovan-Wright et al., 2000) into an *I-SceI* meganuclease vector (Thermes et al., 2002) that also contained *cryaa:mCherry* (Hesselson et al., 2009; Kurita et al., 2003).

To generate the *fabp10a:miR-21SP* plasmid (*fabp10a-miR-21SP-Dendra2*), as outlined by Zhou et al. (2018), we ordered gBlocks^TM^ (IDT, Coralville, Iowa) which contained six bulging miRNA binding sites with MfeI and BamHI restriction sites at the 5′ and 3′ end, respectively (Table S9). gBlocks and *Tol2-lyzC-Vector-Dendra2* were separately digested with MfeI and BamHI, gel extracted, column purified and ligated to generate *Tol2-lyzC-miR-21SP-Dendra2.* From this *Tol2-lyzC-miR-21SP-Dendra2* plasmid, we amplified *miR-21SP* with flanking XhoI restriction sites and placed it downstream of the *fabp10a* promoter (Denovan-Wright et al., 2000) into an *I-SceI* meganuclease vector (Thermes et al., 2002) that also contained *cryaa:mCherry* (Hesselson et al., 2009; Kurita et al., 2003).

One-cell-stage embryos were microinjected with *fabp10a:miR-21* or *fabp10a:miR-21SP* plasmid, *I-SceI* meganuclease, *I-SceI* buffer and Phenol Red as previously described (Thermes et al., 2002). Injected embryos with red eyes and green livers at 2-5 days post fertilization (dpf) were raised to adulthood and crossed to detect founders with germline transmission. We identified three unique miR-21 overexpression (OE) founders (miR-21OE) with similar phenotypes and two unique miR-21 sponge (SP) founders (miR-21SP) with similar phenotypes. The miR-21OE and miR-21SP lines were maintained by outcrossing them to wild-type AB zebrafish each generation. Transgenic zebrafish were distinguished from non-transgenic control siblings by the presence of red eyes and green livers at 3 dpf or later. Phenotypes were consistently seen starting at the F1 generation and all published experiments were performed on F2 generation or later.

### Zebrafish diet studies

For zebrafish larval diet studies, zebrafish were maintained under standard conditions (egg water or low-salt egg water with no feeding) until 6 dpf (Kimmel et al., 1995). From 6 to 12 dpf, zebrafish were fed daily with GEMMA Micro 75 (normal control diet, NCD) or with GEMMA Micro 75 containing 10% by weight cholesterol (C8667, Millipore Sigma), prepared as previously described [high-cholesterol diet (HCD)] (De Oliveira et al., 2019). Zebrafish were maintained in 2.8-l tanks with a total of 1.3 l of low-salt egg water. Water changes were performed daily to remove debris and exchange 1 l of low-salt egg water. At 13 dpf larvae were euthanized and fixed with 4% paraformaldehyde.

For zebrafish adult diet studies, zebrafish were maintained under standard conditions until 8 weeks of age. Beginning at 8 weeks of age, zebrafish were fed twice daily with GEMMA 75, 150, or 500 (NCD) or with GEMMA containing 10% by weight cholesterol, prepared as previously described (HCD) (De Oliveira et al., 2019). Each zebrafish tank was also given 2 ml of suspended brine shrimp once a day. Zebrafish were given a specialized diet for 50 days (7 weeks) and then euthanized ∼12 h after their last feeding.

### Bezafibrate treatment and quantification of larval liver size

The PPAR agonist bezafibrate (MedChemExpress) was dissolved in DMSO and administered to zebrafish larvae in low-salt egg water by continuous immersion. Fish were exposed to 25 µM bezafibrate or DMSO vehicle control (0.1%) from 3 to 6 dpf.

Quantification of larval liver size at 6 dpf was performed as previously described (Kotiyal et al., 2020). In brief, larvae were raised to 6 dpf under standard conditions, euthanized, fixed in 4% paraformaldehyde (PFA), and dissected to remove the pectoral fins, cartilage and skin, to expose the liver and peritoneal cavity. Images were taken of each larva by using a Leica dissecting microscope and analyzed by an examiner unaware of the experimental group using FIJI/ImageJ.

### Quantification of hepatocyte proliferation, nuclear area, inter-nuclear distance and nuclear density

Hepatocyte proliferation was quantified by using the Click-iT Plus EdU Alexa Fluor kit (Thermo Fisher Scientific, C10634) (Evason et al., 2015). To measure *de novo* DNA synthesis 5-ethynyl-2′-deoxyuridine (EdU) from this kit was administered to zebrafish larvae at 20 µM in low-salt egg water by continuous immersion for 3 h directly before euthanasia and fixation in 4% paraformaldehyde. Fish were dissected to expose the liver and peritoneal cavity, the Click-iT reaction was carried out as per the manufacturer's instructions, and DAPI (D1306) counterstain was applied at 300 nM for 3 min. Zebrafish larvae were mounted in 1% agarose plus Slow Fade (P36961).

Samples were imaged on a Leica SP8 Confocal Microscope at 40× magnification, using excitation wave lengths of 405 nm for DAPI and 647 nm for EdU. EdU staining was quantified by manual counting of positive cells in total *z*-stacks of each liver; *z*-stacks varied between 18 and 24 steps per liver based on the size of liver.

DAPI fluorescence images, with one representative optical *z*-slice per larva, were analyzed using QuPath v 0.5.1 (Bankhead et al., 2017). Images were calibrated using confocal metadata (1024×1024 pixels corresponding to 290.62 µm), yielding a pixel size of 0.284 µm/pixel in both the X and Y dimensions. For each image, a polygon annotation was manually drawn to encompass the liver region of interest (ROI). Within each annotated liver ROI, nuclei were identified using QuPath cell detection configured for nuclear-only measurements by setting cytoplasmic expansion to 0 µm. This approach allowed extraction of nuclear counts, nuclear area and nuclear centroid coordinates based exclusively on DAPI signal, without inclusion of cytoplasmic measurements. Nuclear density was calculated as the number of nuclei per unit area (nuclei per 1000 µm^2^). Estimated inter-nuclear spacing was calculated in Excel using centroid-based distance metrics derived from the detected nuclei, providing an indirect measure of relative hepatocyte spacing and cell size.

### Quantification of steatosis

Fixed 13 dpf larvae were stained with Oil Red O (ORO) by washing 4% PFA-fixed fish in isopropanol for 30 min followed by addition of fresh isopropanol with 0.3% dissolved ORO. The fish were left to rock on orbital rocker for 90 min in this solution and washed once with isopropanol then PBS for 5 min each on orbital rocker (Wilson et al., 2020). Within 48 h, the larvae were dissected to expose the liver and imaged with a Leica dissecting microscope. Masked images were given a semi-quantitative score based on relative ORO staining intensity on a scale from 0 (no staining) to 4 (complete, intense staining).

Nile Red (Sigma, 72485) was used to stain intracellular lipid droplets in 6 dpf zebrafish larvae to quantify steatosis by confocal imaging. Staining protocol was done according to Khan et al. (Khan et al., 2024). Fish were collected after completion of the larval HCD. Fish were exposed to Nile Red at a final concentration of 500 ng/ml for 1 h at 28°C covered with foil to shield from light. After Nile Red exposure fish were euthanized and fixed with 4% paraformaldehyde. After being in fixative for 24 h the fish were deskinned and mounted in low melt agarose for imaging. Fish were imaged at 488 nm and 526 nm (Khan et al., 2024) to visualize the amount of phospholipid and lipid droplet accumulation, respectively. Green lipid droplets were calculated for the entire liver. To include red lipid droplets, each image was cropped to a 250×250 pixel area of the liver to eliminate signal from outside of the liver. Analysis was completed using ImageJ. One-way ANOVA was used for statistical analysis.

For adult zebrafish, frozen sections and ORO staining were performed by ARUP Research Histology. Slides were examined by an examiner unaware of the experimental groups, and one representative image was taken of each slide using an Olympus BX53 microscope with Olympus DP73 camera and cellSens software. Within each image the area stained red was quantified with FIJI/ImageJ. One-way ANOVA was used for statistical analysis.

### RNA sequencing and qRT-PCR

Zebrafish at 13 dpf were euthanized, and livers were dissected and pooled (13 livers per sample) for RNA extraction using the PicoPure RNA extraction kit (Thermo Fisher Scientific, KIT0204). Each sample comprised pooled livers from the same clutch of zebrafish. For sequencing, libraries were prepared by the HTG Core using NEBNext Ultra II Directional RNA Library Prep with rRNA Depletion Kit (Zebrafish). Samples were barcoded, pooled and sequenced using paired 150 bp sequencing on Illumina NovaSeq X. Reads were aligned to GRCv11 zebrafish genome. Genes with less than ten counts were removed. The initial experiment included four experimental groups (WT-NCD, WT-HCD, miR21OE-NCD and miR21OE-HCD). There were three samples in each experimental group.

Differentially expressed transcripts were identified with DESeq2 version 1.40.2 (Love et al., 2014) and were considered significantly dysregulated with padj<0.05. Pathway analysis was completed using ShinyGO v0.85.1 either using all dysregulated genes, or specifically only those down or upregulated (Ge et al., 2020). FGSEA v 1.36.0 was used on the C3 TFT motifs with 20% false discovery rate, to determine if there was transcription factor enrichment (Korotkevich et al., 2021 preprint). TargetScanFish v6.2 (Horton et al., 2002; Jagtap et al., 2025; Janssen et al., 2013) was accessed on 10 December 2025 to identify predicted miR-21 seed sequences (3590 predicted genes) and cross referenced with the 67 downregulated miR-21 dependent genes (Table S3 Workbook S3g) with 23 genes identified (Table S3 Workbook S3i). Ulitsky et al. (2012) found that 20,988 zebrafish genes have 3′ UTR >10 bp, indicating that 17% of zebrafish genes (3590 predicted genes with miR-21 seed sequences out of 20,988 genes) contain miR-21 binding sites. Fisher's exact test (GraphPad Prism) was used to determine the significance.

To compare gene expression changes in MASLD for mouse and zebrafish, a mouse (GSE230639) (Tsouka et al., 2024) dataset was accessed using GEOquery v2.78.0 (Davis and Meltzer, 2007) to compare non-tumorous DIO tissue to chow controls (Table S5 Workbook S5d). We analyzed an expanded dataset of WT-NCD and WT-HCD samples, which for each experimental group included the three samples from the original experiment plus 6 additional samples. Differentially expressed transcripts were identified with DESeq2 version 1.40.2 (Love et al., 2014) and were considered significantly dysregulated with padj<0.05 (Table S5 Workbooks S5a,b). Homology with zebrafish was identified using Ensembl BiomaRt v2.66.0 (Durinck et al., 2005, 2009) with 30% identity, evaluating 9769 genes (Table S5 Workbook S5c). Prior data sets of ABC zebrafish for male (Kalasekar et al., 2019) and female (Evason et al., 2015) were also used. Overlap comparison between datasets was performed with Fisher's exact test for two datasets or SuperExactTest v1.1 (Wang et al., 2015) for more than two datasets.

Samples for qRT-PCR were reverse transcribed using Super Script III kit (catalog number 18080051). Primer sequences are listed in Table S10. qRT-PCR master mixes were prepared consisting of 2.5% 100 μM forward primer, 2.5% 100 μM reverse primer and 62.5% PowerTrack SYBR Green Master Mix (Thermo Fisher Scientific, A46109) in RNase-free water. Master mixes were combined 4:1 with the cDNA reactions and plated in duplicate. qRT-PCR was performed using the LC480 PCR Lightcycler (Roche, 05015278001) using the ‘Mono Color Hydrolysis Probe/UPL probe’ detection format. The temperature cycle consisted of an initial 2 min period at 95°C and 40 cycles of 95°C for 15 s and 60°C for 50 s set to single acquisition mode. The housekeeping gene β-actin was used as an internal control for cDNA quantification and normalization of amplified products. Data are reported as relative expression.

### Histological evaluation of H&E-stained zebrafish tissue sections

Whole-body paraffin embedding, sectioning and staining with Hematoxylin and Eosin (H&E) was performed by ARUP Research Histology. Sections were reviewed and scored by a board-certified pathologist (K.J.E.) in a masked manner using an Olympus BX53 microscope. For larval zebrafish (13 dpf), one to three histologic sections of liver were examined for each zebrafish. Steatosis was scored by estimating the percentage of liver parenchyma involved by fat (clear, circular intracellular spaces with smooth contours) across all sections. Inflammation was scored by counting the number of inflammatory clusters, defined as a group of five or more inflammatory cells, and by dividing that number by the number of sections examined. For adult zebrafish, at least one liver section was examined. Each zebrafish was assigned to one of the following categories as previously described (Evason et al., 2015): (1) no changes, defined as no substantial cytological or architectural abnormalities; (2) mild changes, defined as the presence of cytological abnormalities in the absence of substantial architectural abnormalities or vice versa; (3) hepatocellular carcinoma (HCC), defined as the presence of both architectural and cytological abnormalities; and (4) cholangiocarcinoma (CCA), defined as the presence of cytologically abnormal cells forming irregular glands, surrounded by dense stroma. These terms were given a numerical score between 0 and 4, respectively, and then graphed and analyzed using GraphPad Prism.

### Lipid extraction

Adult zebrafish were grouped based on diet, sex and genotype. Livers were isolated using a dissecting microscope and weighed. Livers were snap frozen in liquid nitrogen and placed on dry ice. For male samples, liver tissues from up to three animals of the same genotype/diet group were pooled to achieve a minimum of 15 mg. Female samples had sufficient tissue of >15 mg and were unpooled. We analyzed between one and six replicates per group.

10-30 mg of flash-frozen zebrafish liver tissue were isolated in 2 ml Safelock microcentrifuge tubes (Eppendorf, 022363352) with a 5/16 in. diameter stainless steel ball (Grainger, 4RJL8) chilled to −80°C. Tissue was homogenized at 25 Hz for 30 s under liquid nitrogen using a Retsch Cryomill (Retsch, 20.749.0001). Lipids were extracted by adding 1 ml of 75% methyl tert-butyl ether, 24% µl methanol and 1% Splash Lipidomix Mass Spec Standard (Avanti Polar Lipids 330707) and incubating on ice for 15 min with intermittent vortexing; phase separation was induced with 190 µl ultrapure water. The organic supernatant was transferred to a glass vial and dried with gaseous nitrogen. Analytes were resuspended in 25 µl/mg sample in 2:1:1 isopropyl alcohol:acetonitrile:water and transferred to amber mass spectrometry vials (Agilent, 5182-0716) with glass inserts.

### LC-MS lipidomics

Lipid extracts were analyzed by LC-MS using a Vanquish HPLC system (Thermo Fisher Scientific) and a QExactive HF Orbitrap mass spectrometer (Thermo Fisher Scientific). Separation was achieved by C18 chromatography performed on an Acquity UPLC CSH C18 column (2.1 mm×100 mm, 1.7 µm particular size, 130 Å pore size, Waters Co., 186005297). The chromatography gradient was formed by solvent A (10 mM ammonium formate and 0.1% formic acid in 60:40 acetonitrile:water) and solvent B (10 mM ammonium formate and 0.1% formic acid in 90:9:1 isopropyl alcohol:acetonitrile:water) at a constant flow rate of 350 µl/min. The gradient function was: 0 min, 30% B; 5 min, 43% B; 5.1 min, 50% B; 14 min, 70% B; 21 min, 99% B; 24 min, 99% B; 24.1 min, 30% B; 31 min, 30% B. Autosampler temperature was 4°C, column temperature was 30°C and injection volume was 2 μl. Samples were injected into the mass spectrometer by electrospray ionization operating in positive ion mode. Lipid mass spectra were collected in full scan mode at 70,000 resolving power, and peaks were identified based on exact mass and retention times using El-MAVEN with comparison with known standards (Clasquin et al., 2012).

### Statistics

GraphPad Prism V10.2 was used to perform statistical analyses and generate graphs. For larval liver size, liver-to-body ratio and other continuous data, we performed ordinary one-way ANOVA with Sidak's multiple comparisons tests or, if standard deviations were not equal, Brown-Forsythe and Welch ANOVA tests with Dunnett's multiple comparisons test. For ORO staining, histological diagnosis and other ordinal/semi-quantitative data, we performed Kruskal–Wallis tests with Dunn's multiple comparisons test. For RNA sequencing, differential expression was identified with DESeq2 version 1.40.2 (Love et al., 2014). Pathway analysis was performed with ShinyGO v0.85.1 (Ge et al., 2020) and overlap comparison between datasets was performed with Fisher's exact test for two datasets or SuperExactTest v1.1 (Wang et al., 2015) for more than two datasets. For lipidomics comparison a two-way ANOVA with Dunnett post hoc analysis was used for comparing genotype effects. To test diet effect across genotypes, two-way ANOVA with Šidák post-hoc analysis was used.

## Supplementary Material

10.1242/dmm.052583_sup1 Supplementary information

Table S2. miRNA analysis

Table S3. miR-21 and HCD RNAsequencing results

Table S4. Shiny Go

Table S5. Zebrafish compared to DIO mouse

Table S6. C3 TFT GSEA

Table S7. miR21 and ABC ZF
