# Supplementary material for: MicroRNA-21 promotes dysregulated lipid metabolism and hepatocellular carcinoma
Source: Dis Model Mech. 2026 Mar 5;19(2):dmm052583. doi: 10.1242/dmm.052583 (PMC12994464; doi:10.1242/dmm.052583)
Supplement: Supplementary information [file dmm-19-052583-s1.pdf]

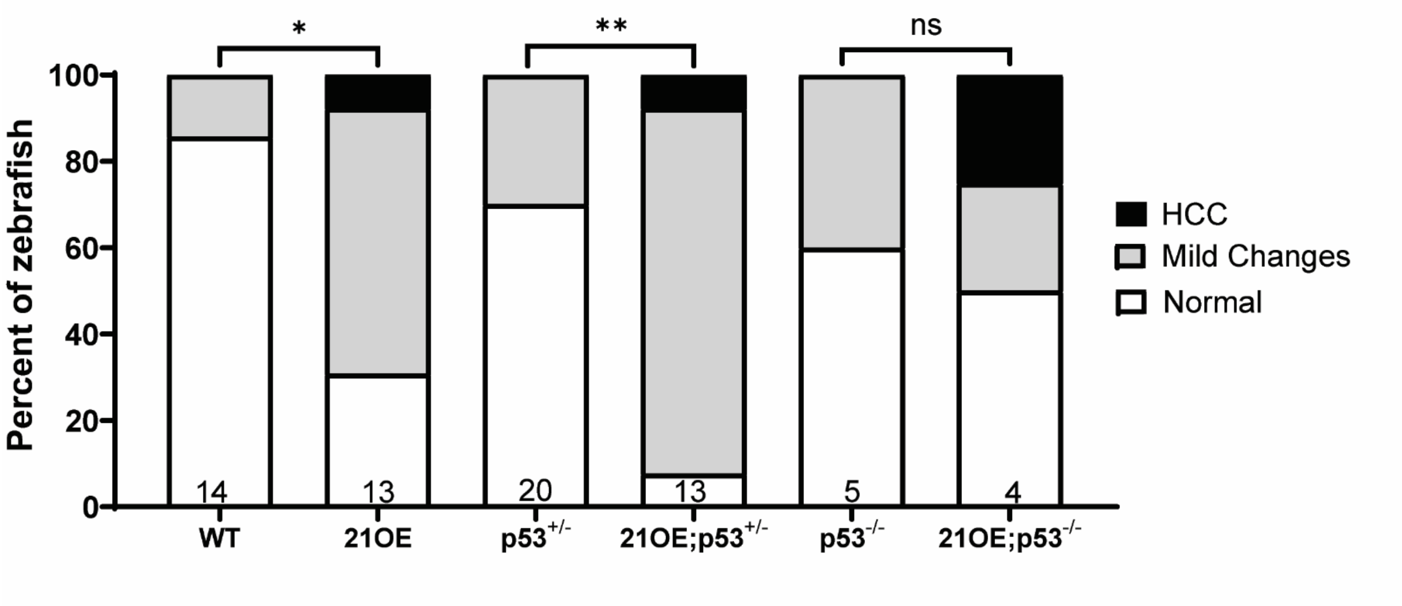

Fig. S1. miR-21 causes mild changes and HCC in adult zebrafish. Histology of miR-21OE (21OE) and non-transgenic wildtype sibling (WT) adult zebrafish livers (12 mpf) in the presence of p53 homozygous (p53- /-) or heterozygous (p53+/-) mutations was assessed by hematoxylin and eosin (H&E) stain. P values determined with GraphPad Prism, Kruskal-Wallis test with Dunn's multiple comparisons test: ns, not significant; *, $p < 0.05$; **, $p < 0.01$. N values are indicated at the bottom of each column.

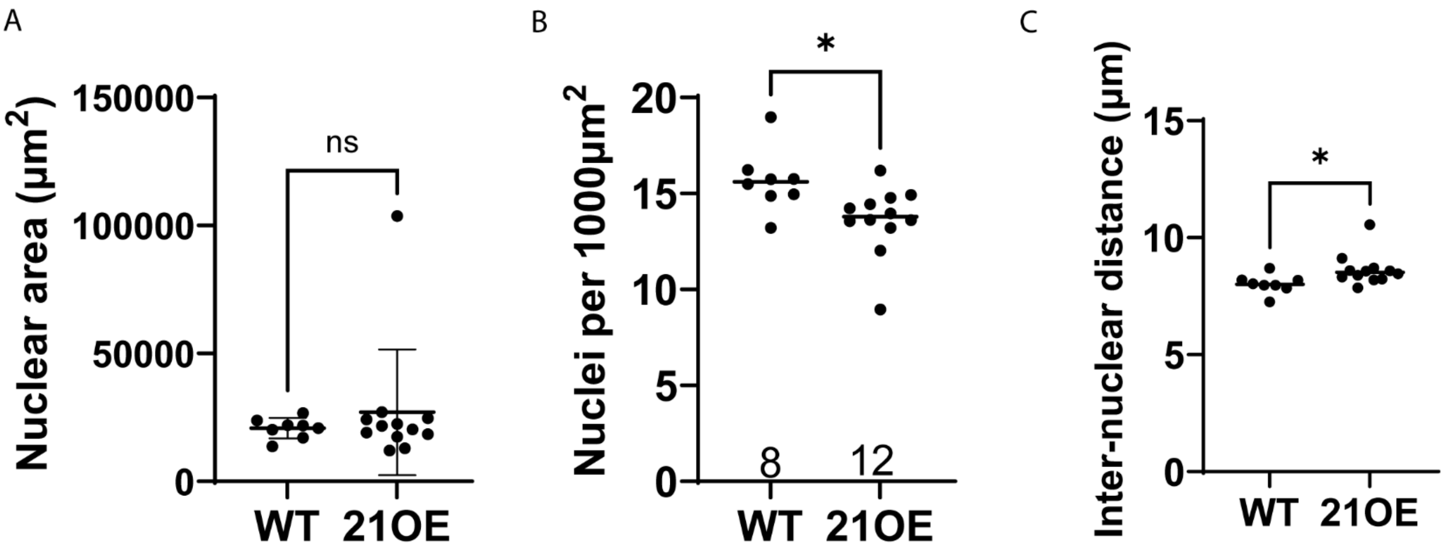

Fig. S2. miR-21 decreases nuclear density and increases inter-nuclear distance. Confocal images of DAPI-stained non-transgenic wildtype control sibling (WT) and miR-21OE liver at 6 dpf were analyzed with QuPath. N values are shown above the x axis in panel B. P values determined with GraphPad Prism using Welch's t test (A) or unpaired t test (B, C): ns, not significant; *, $p < 0.05$. The experiment was performed twice with similar results each time, and one representative experiment is shown.

Disease Models & Mechanisms • Supplementary information

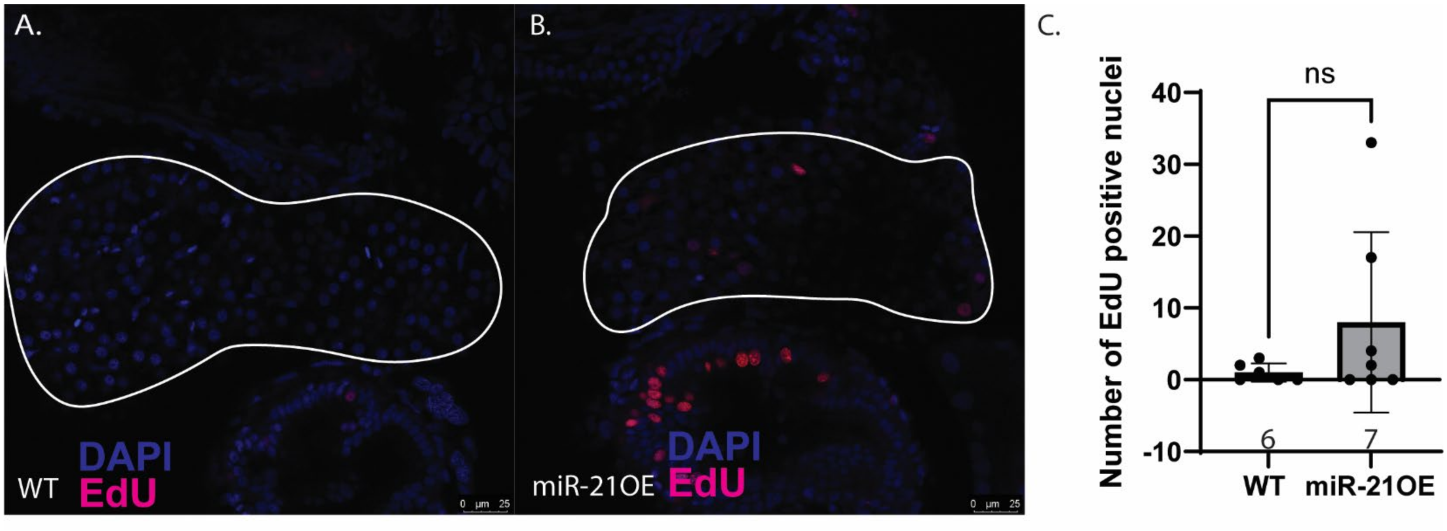

Fig. S3. miR21OE does not significantly alter EdU labeling. A-B. Representative images of non-transgenic wildtype control sibling (WT) and miR-21OE liver at 6 dpf. Livers are outlined in white. C. Counts of EdU-positive nuclei in 6 dpf livers. N values are above the x axis. Mann-Whitney test was performed with GraphPad Prism: ns, not significant. The experiment was performed three times with similar results each time.

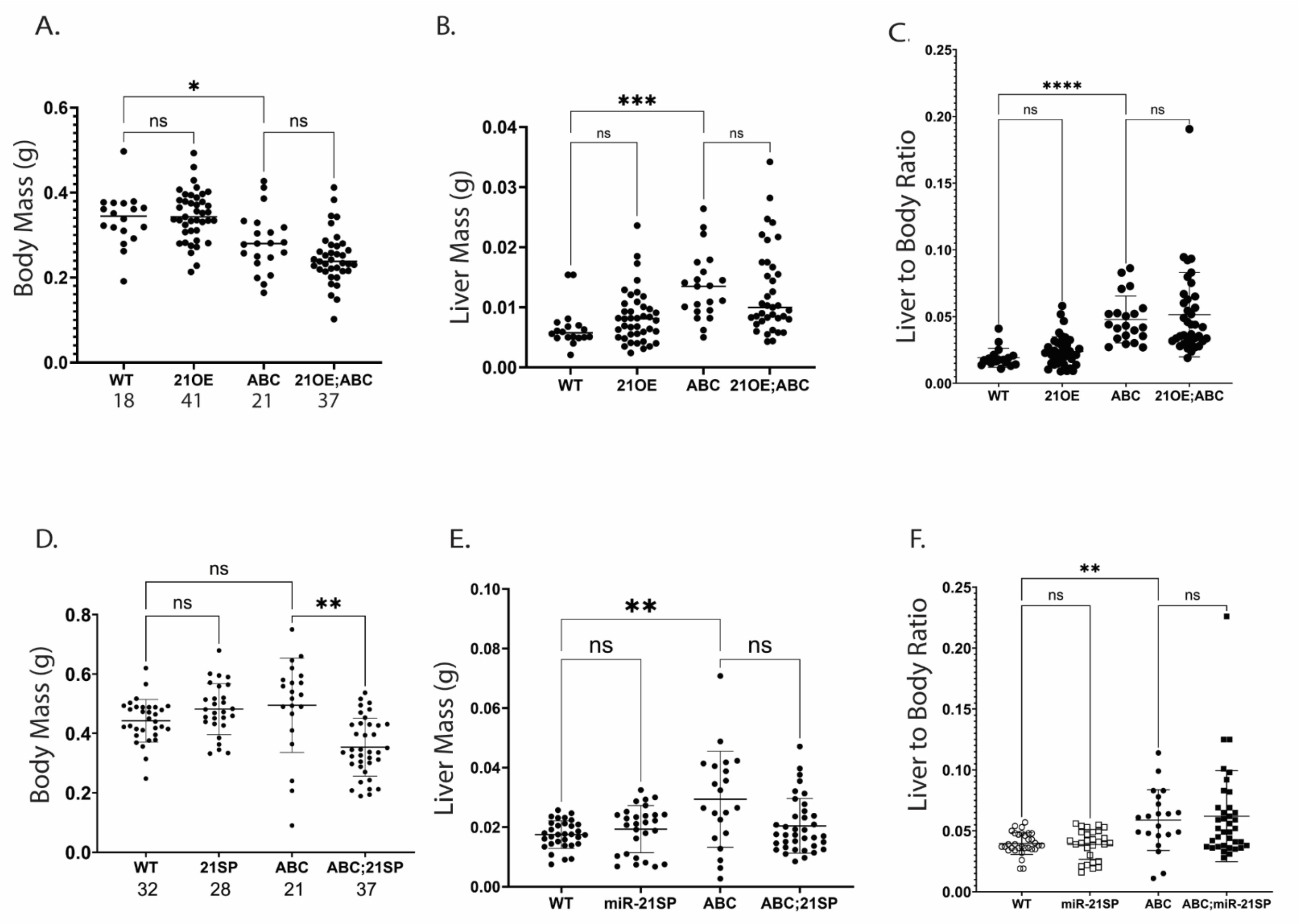

Fig. S4. miR-21OE and miR-21SP do not significantly alter liver weight or body weight. A-C. We raised miR-21OE (21OE), ABC, 21OE;ABC, and non-transgenic wildtype control siblings (WT) under standard conditions to 10 weeks of age. After euthanizing zebrafish, we determined body mass (A), liver mass (B), and liver-to-body ratios (C). D-F. We raised miR-21SP (21SP), ABC, 21SP;ABC, and non-transgenic wildtype control siblings (WT) under standard conditions to 10 weeks of age. After euthanizing zebrafish, we determined body mass (D), liver mass (E), and liver-to-body ratios (F). N values for each of the two experiments are shown below the x-axis in panels (A) and (D). GraphPad Prism (Brown-Forsythe and Welch ANOVA followed by Dunnett's T3 multiple comparisons test) was used to determine P-values: ns, not significant; *, $p < 0.05$; **, $p < 0.01$; ***, $p < 0.001$; ****, $p < 0.0001$.

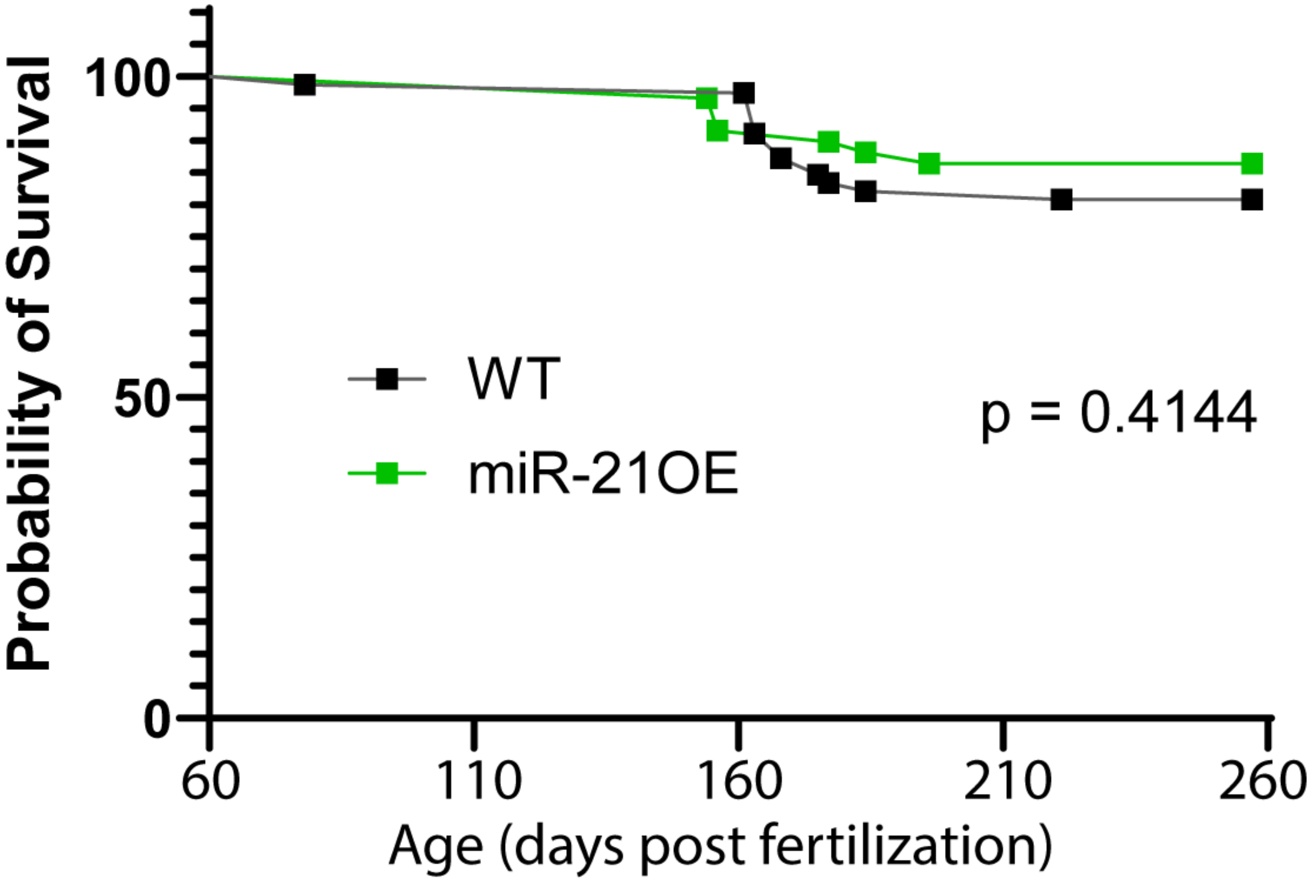

Fig. S5. miR-21OE does not significantly impact survival. Three tanks (59 zebrafish) of miR-21OE zebrafish (miR-21OE) and four tanks (78 zebrafish) of non-transgenic wildtype control siblings (WT) were monitored for survival at least three times per week starting at 2 months of age (60 dpf). P-value determined with GraphPad Prism, Log-rank (Mantel-Cox) test.

Disease Models & Mechanisms • Supplementary information

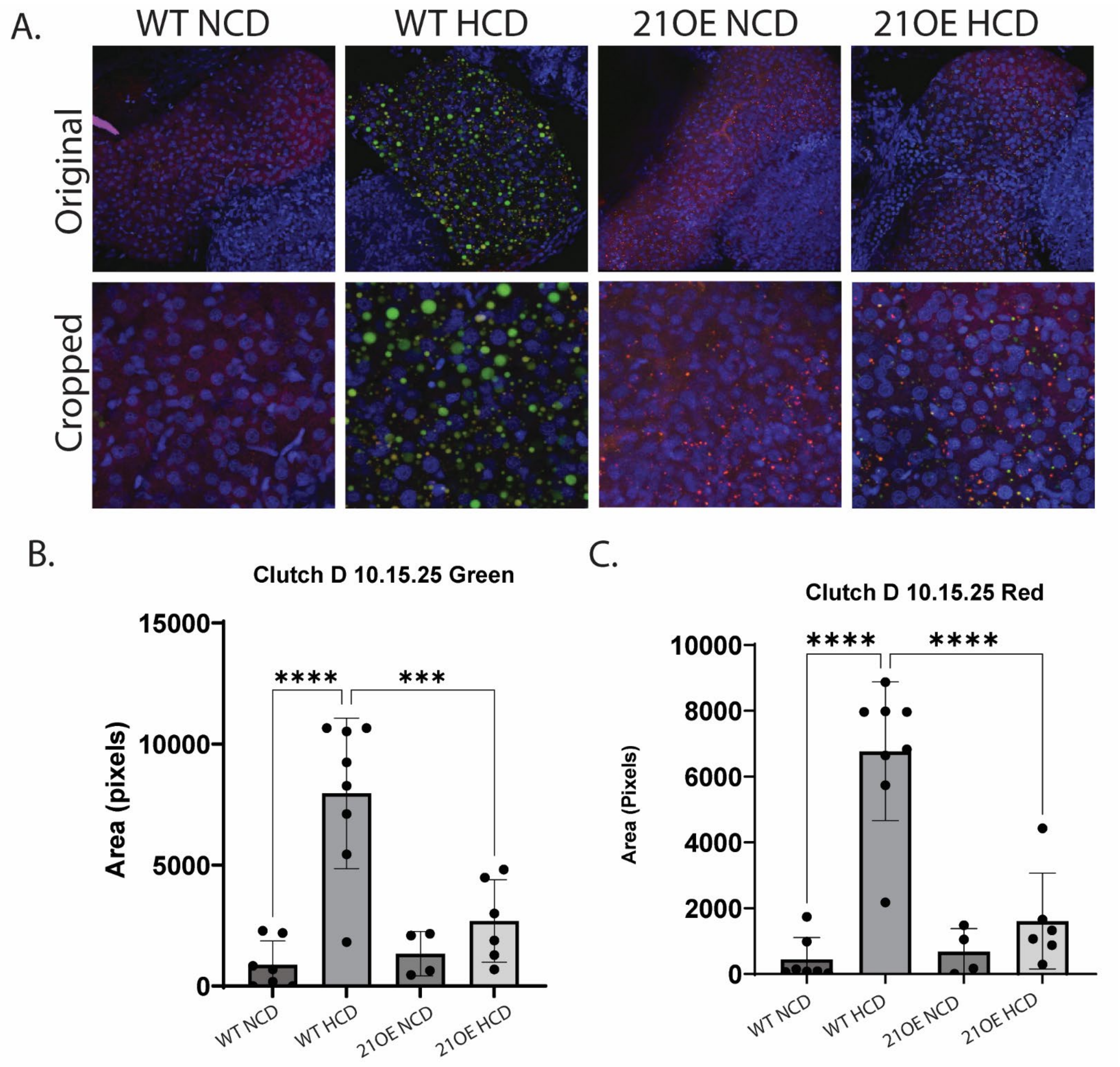

Fig. S6. Quantification of Nile Red staining. A. Representative confocal images from Nile Red Stain showing full uncropped image of entire liver on top and cropped image used for analysis directly below. B-C. Quantification of Nile Red stain on green (B) and red (C) channels. N values were as follows: WT NCD = 7; WT HCD = 8; 21OE NCD = 4; 21OE HCD = 6. P values determined with GraphPad Prism, One way ANOVA: ns, not significant; ***, $p < 0.001$; ****, $p < 0.0001$. The experiment was performed three times with similar results each time; one representative experiment is shown here.

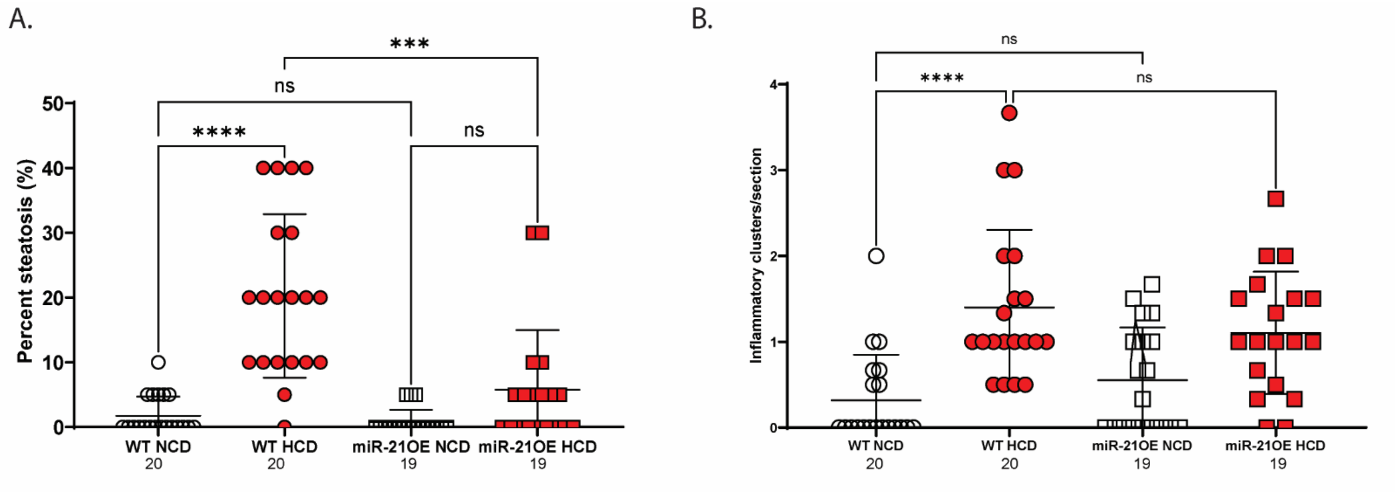

Fig. S7. miR-21OE decreases steatosis but does not affect inflammation at 13 dpf as assessed by hematoxylin and eosin (H&E) staining. A. Steatosis was quantified by pathologist (KJE) assessment of H&E-stained slides in a masked fashion. B. For each zebrafish, we counted the number of inflammatory clusters--groups of 5 or more inflammatory cells--in the liver and normalized to the number of histologic sections examined. One to three sections were examined for each zebrafish. P values were determined with GraphPad Prism, Kruskal-Wallis test with Dunn's multiple comparisons test: ns, not significant; ***, $p < 0.001$; ****, $p < 0.0001$. The experiment was performed three times with similar results each time; one representative experiment is shown here.

Disease Models & Mechanisms • Supplementary information

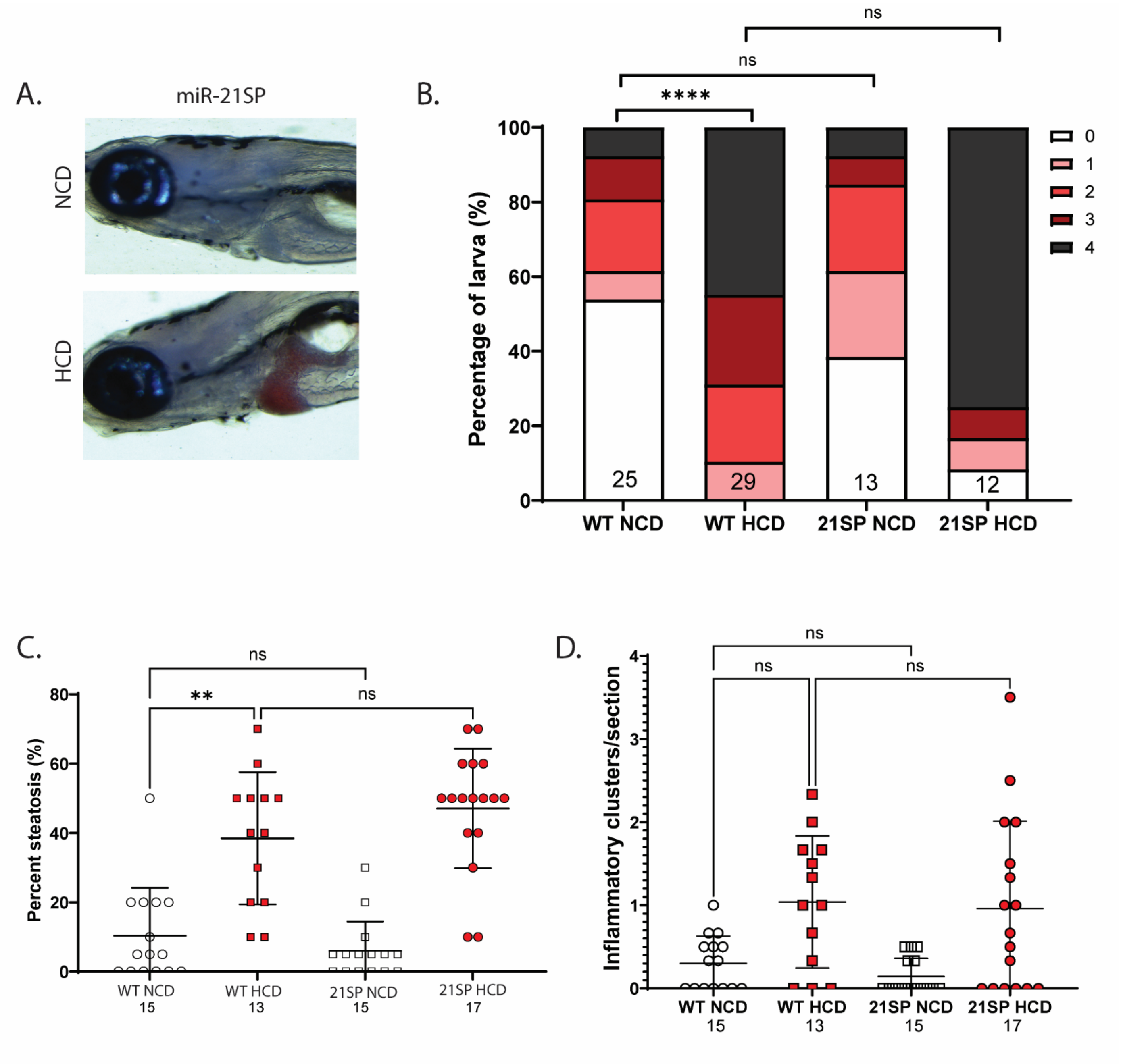

Fig. S8. miR-21SP does not affect steatosis or inflammation at 13 dpf. A. Representative brightfield images of 13-dpf zebrafish after Oil Red O staining. B. Quantification of ORO of larval liver images. Scoring is from 0-4. N values are indicated at the bottom of each column. P values were determined with GraphPad Prism, Kruskal-Wallis test with Dunn's multiple comparisons test: ns; not significant; ****, $p < 0.0001$. C-D. Steatosis (C) and inflammation (D) were quantified by pathologist (KJE) assessment of H&E-stained slides in a masked fashion. N values are shown below the x axis. P values were determined with GraphPad Prism, Kruskal-Wallis test with Dunn's multiple comparisons test: ns, not significant; **, $p < 0.01$. Each experiment was performed three times with similar results each time; one representative experiment is shown here.

Disease Models & Mechanisms • Supplementary information

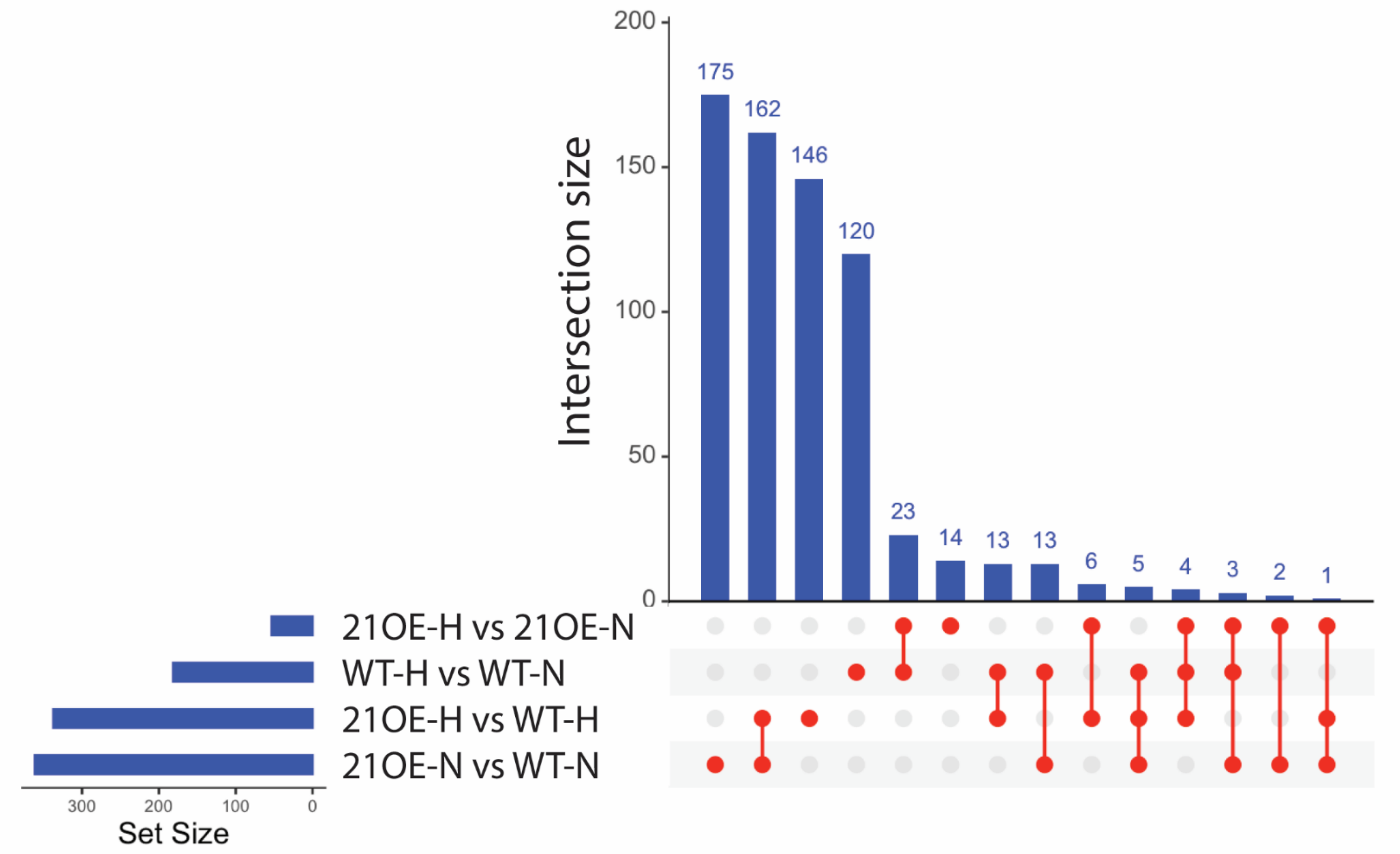

Fig. S9. Changes across genotype and diet. UpSet Plot of RNA-seq data showing number of altered genes across comparisons for miR-21OE (21OE) and non-transgenic wildtype sibling control (WT) livers from 13-dpf zebrafish fed high cholesterol diet (H) or normal control diet (N).

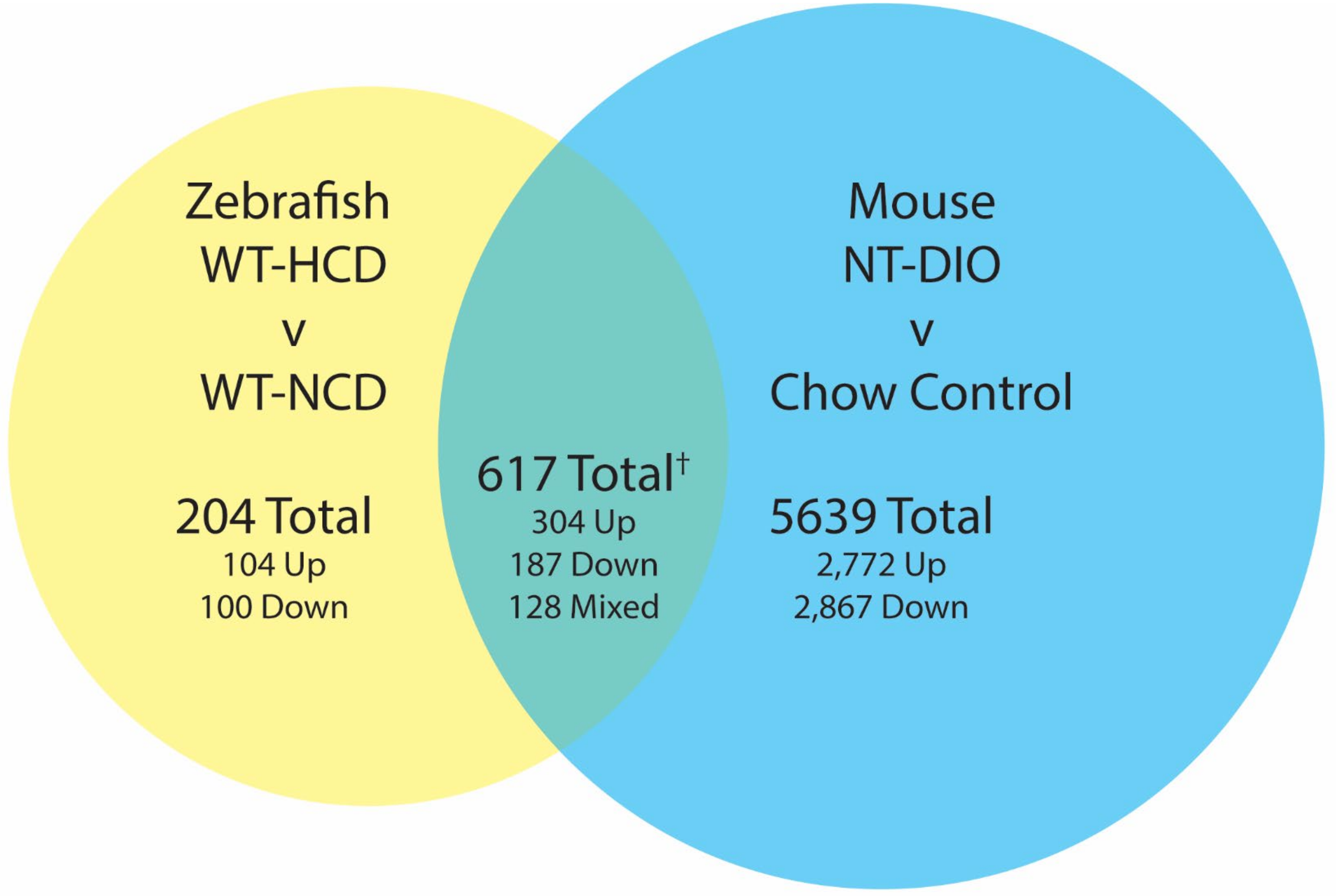

Fig. S10. Comparison of zebrafish and mouse MASLD models. Venn diagram showing overlapping dysregulated genes in zebrafish (non-transgenic wildtype zebrafish (WT) fed high cholesterol diet (HCD) versus normal control diet (NCD)) and mouse (non-tumor diet-induced obesity (NT-DIO) versus chow control (Workbook S5d) (GSE243976). Zebrafish dataset consisted of 9 samples each of WT-HCD and WT-NCD, all sequenced on the same run (Workbook S5a). NT-DIO v Chow (Workbook S5b). Gene names are shown in Workbook S5c. †, p = 7e-13, Fisher's exact test, GraphPad Prism.

Disease Models & Mechanisms • Supplementary information

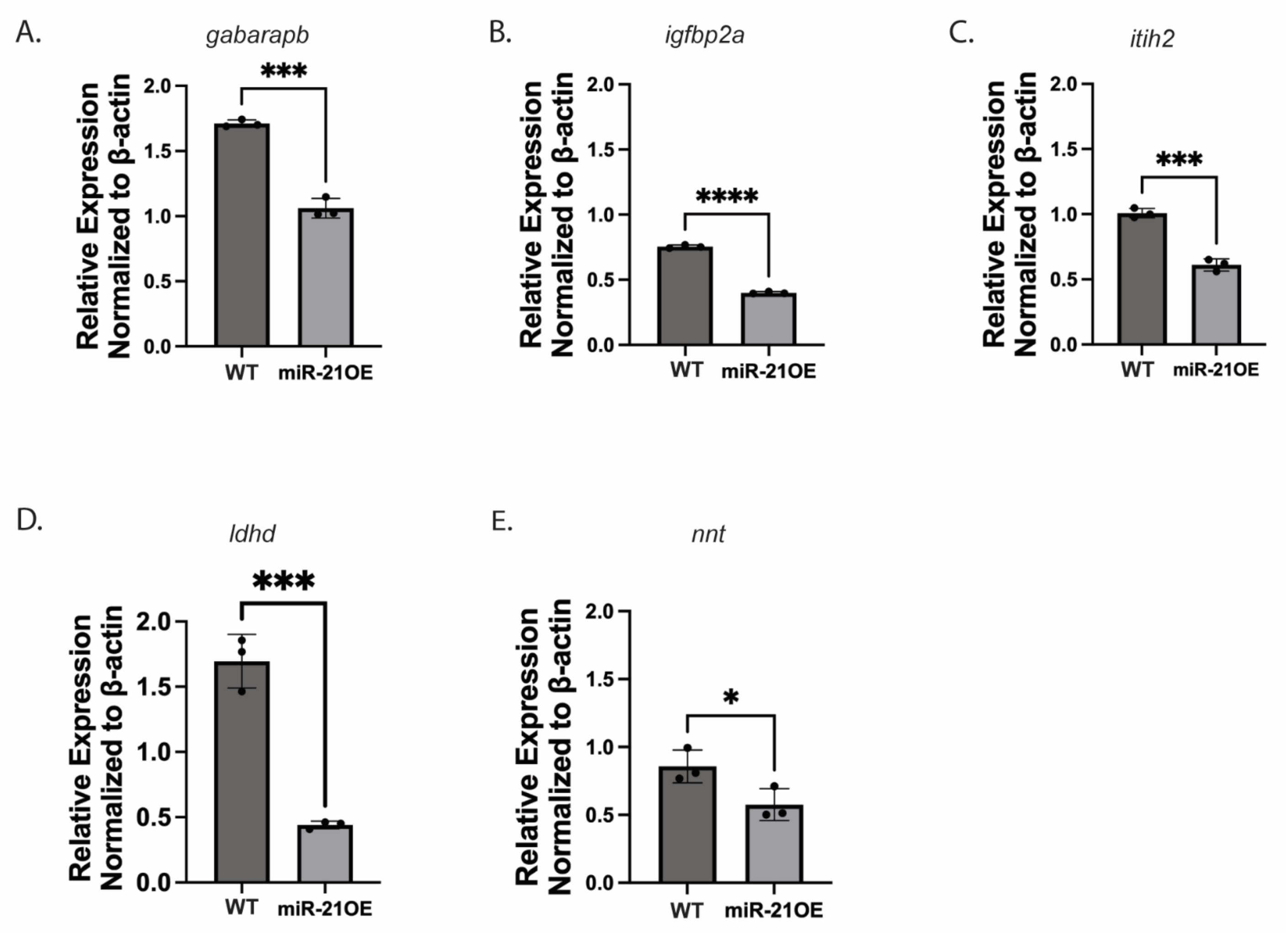

Fig. S11. qRT-PCR of genes in miR-21OE (21OE) and controls (W) relative to β-actin. We confirmed expression of select genes by performing qRT-PCR for three independent experiments (3 clutches) of 13-dpf miR-21OE zebrafish and non-transgenic wildtype control siblings (WT) fed a normal control diet. One representative experiment (with three technical replicates) is shown for each gene: *gabarapb* (A), *igfbp2a* (B), *itih2* (C), *ldhd* (D), and *nnt* (E). P values were determined with GraphPad Prism, Students T-Test: *, $p < 0.05$; **, $p < 0.01$; ***, $p < 0.001$; ****, $p < 0.0001$.

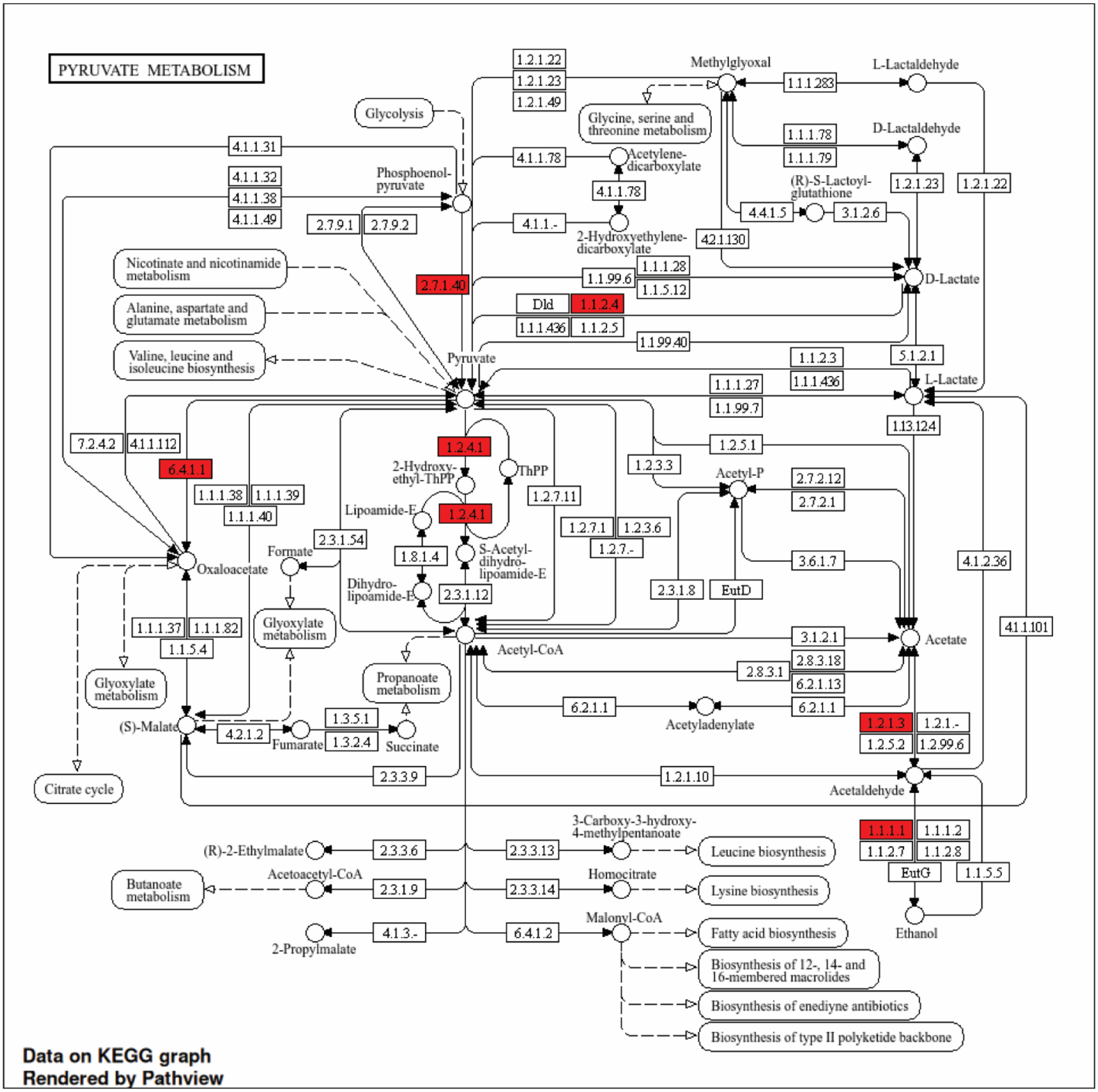

Fig. S12. Visual map of KEGG Pathway “pyruvate metabolism” (dre00620), which was enriched in the 67 downregulated, miR-21 dependent genes (6 genes identified, 49.1 fold enrichment, FDR=1.5e-7) (Workbook S4j). Ensembl IDs of the 67 down regulated miR-21 dependent genes (Workbook S3g) were used with ShinyGO v0.85.1 set to D. rerio and an FDR cutoff of 0.05. Pathway map was rendered via ShinyGO with identified genes in pathway highlighted red.

Disease Models & Mechanisms • Supplementary information

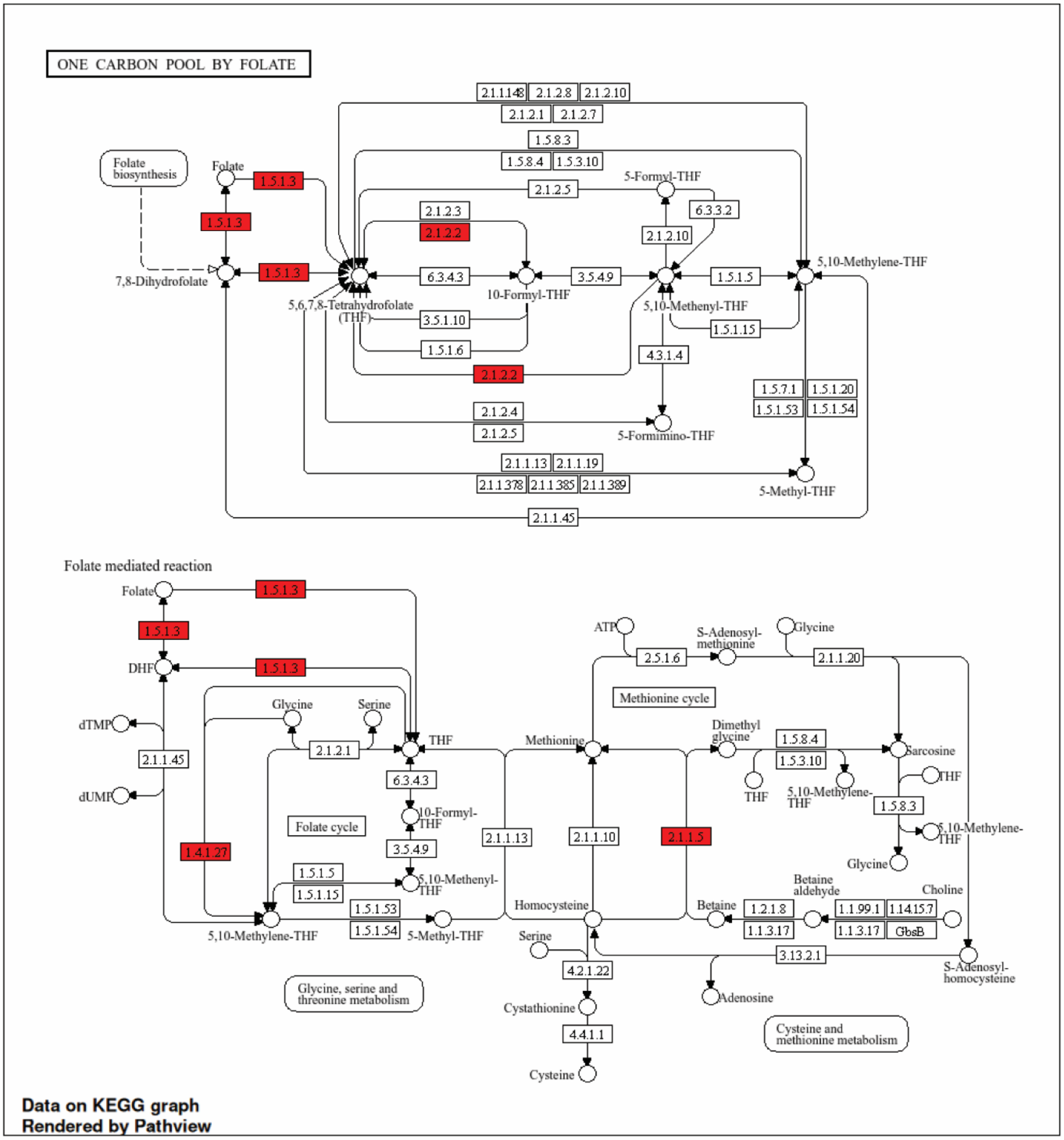

Fig. S13. Visual map of KEGG Pathway "one carbon pool by folate" (dre00670) was enriched in the 95 upregulated, miR-21 dependent genes (4 genes identified, 30.2 Fold Enrichment, FDR=7.4E-4) (Workbook S4k). Ensembl IDs of the 95 upregulated genes (Workbook S2g) were used with ShinyGO v0.85.1 set to D. rerio and an FDR cutoff of 0.05. Pathway map was rendered via ShinyGO with identified genes in pathway highlighted red.

Disease Models & Mechanisms • Supplementary information

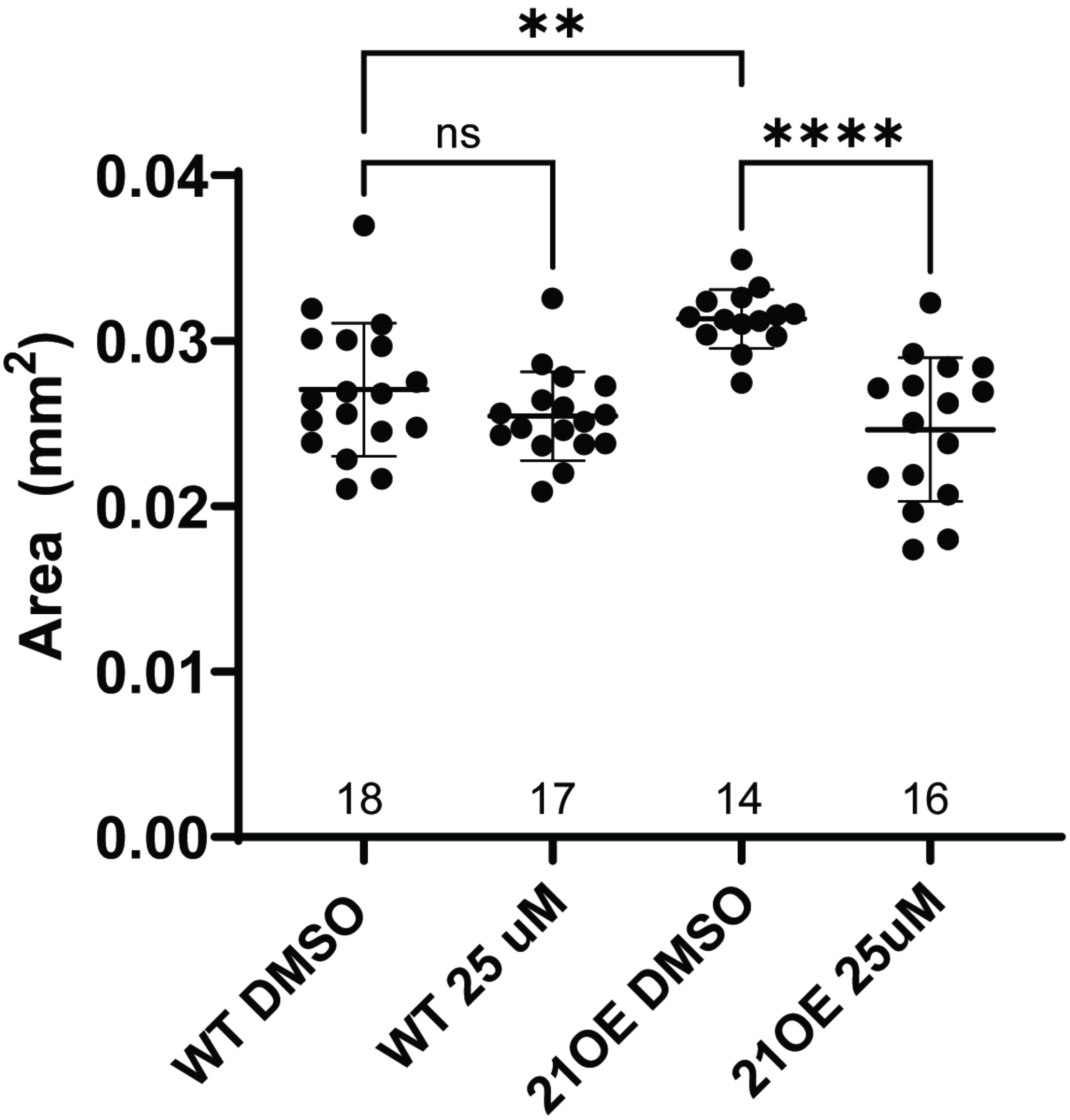

Fig. S14. PPARα agonist bezafibrate decreases miR-21OE-driven larval liver overgrowth. Liver area at 6 dpf of miR-21OE zebrafish (21OE) and non-transgenic wild-type siblings (WT) treated with vehicle (DMSO) or 25 μM bezafibrate. N values are shown above the x axis. P values were determined with GraphPad Prism (Brown-Forsythe and Welch ANOVA tests with Dunnett's T3 multiple comparisons test: ns, not significant; **, p < 0.01; ****, p < 0.0001. Two independent replicates of this experiment were performed with similar results, and one representative experiment is shown.

Disease Models & Mechanisms • Supplementary information

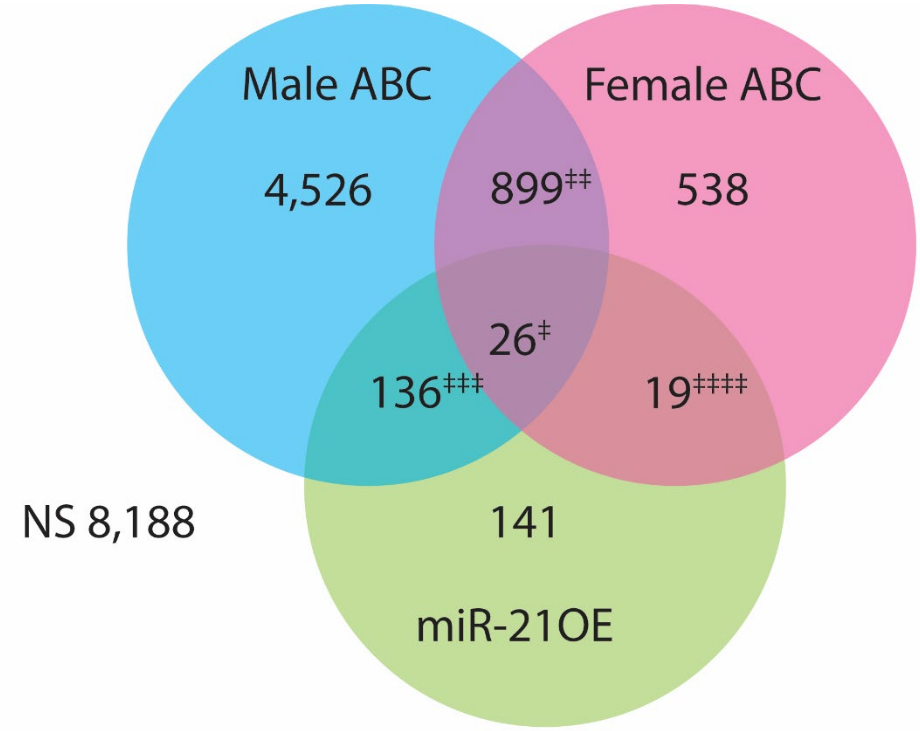

Fig. S15. There is significant overlap between miR-21OE-NCD v WT-NCD (Workbook S3b), female adult ABC zebrafish, and male adult ABC zebrafish datasets using SuperExactTest. Genes identified in all three datasets are listed in Workbook S7a. These data were then compared with SuperExactTest and identified significant overlap between each comparison (Workbook S7b, ‡p=4.7e-4, ‡‡p=6.14e-86, ‡‡‡p=4.91e-6, ‡‡‡‡p=1.88e-2). Note, Workbook S7b comments on a total of three genes, which were analyzed with two Ensembl IDs each aligning to one gene name. A total of 26 genes were identified as signficantly dysregulated in each of the three datasets and are listed in Workbook S7c.

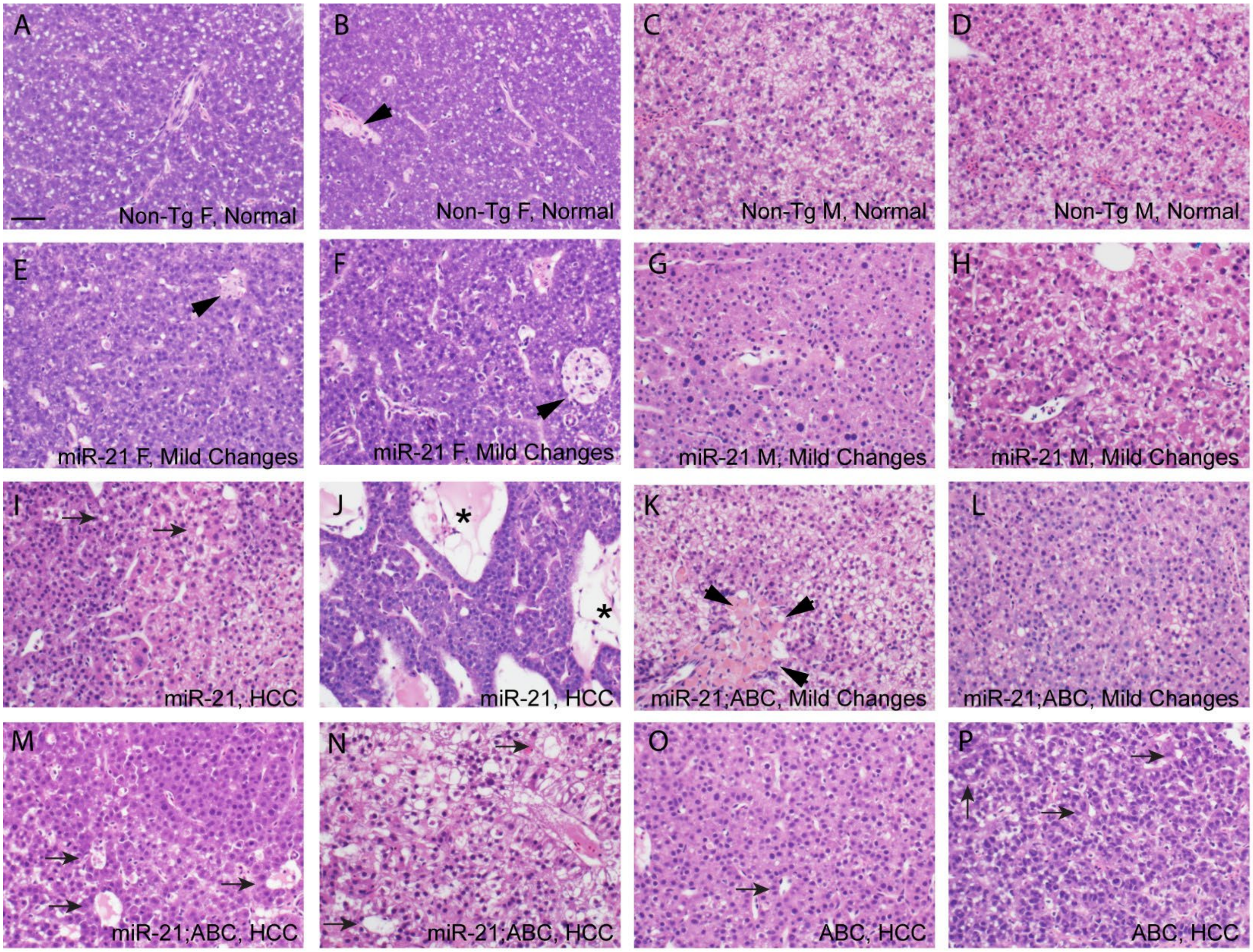

Fig. S16. Representative images of adult zebrafish livers. Scale bar is 20 microns. (A-D) Non-transgenic female (A, B) and male (C, D) zebrafish showing no significant pathologic abnormalities (no tumor, normal). High cholesterol diet (B) did not result in any obvious changes in cytoplasmic droplets compared to normal control diet (A, C, and D); it is difficult to distinguish glycogen from fat on hematoxylin and eosin-stained slides. (E-J) miR-21 female (E, F, J) and male (G, H, I) zebrafish showing mild changes (E-H), characterized by mild nuclear enlargement and/or nuclear contour irregularities without significant architectural abnormalities, or HCC (I, J), characterized by both cytologic and architectural abnormalities. (K-N) miR-21;ABC zebrafish showing mild changes (K, L) or HCC (M, N). (O,P) ABC zebrafish showing HCC. We occasionally noted clusters of inflammatory cells in zebrafish administered a high cholesterol diet (arrowheads). Architectural abnormalities seen in HCC included pseudogland formation (arrows) and peliosis hepatis-like change (asterisks).

Disease Models & Mechanisms • Supplementary information

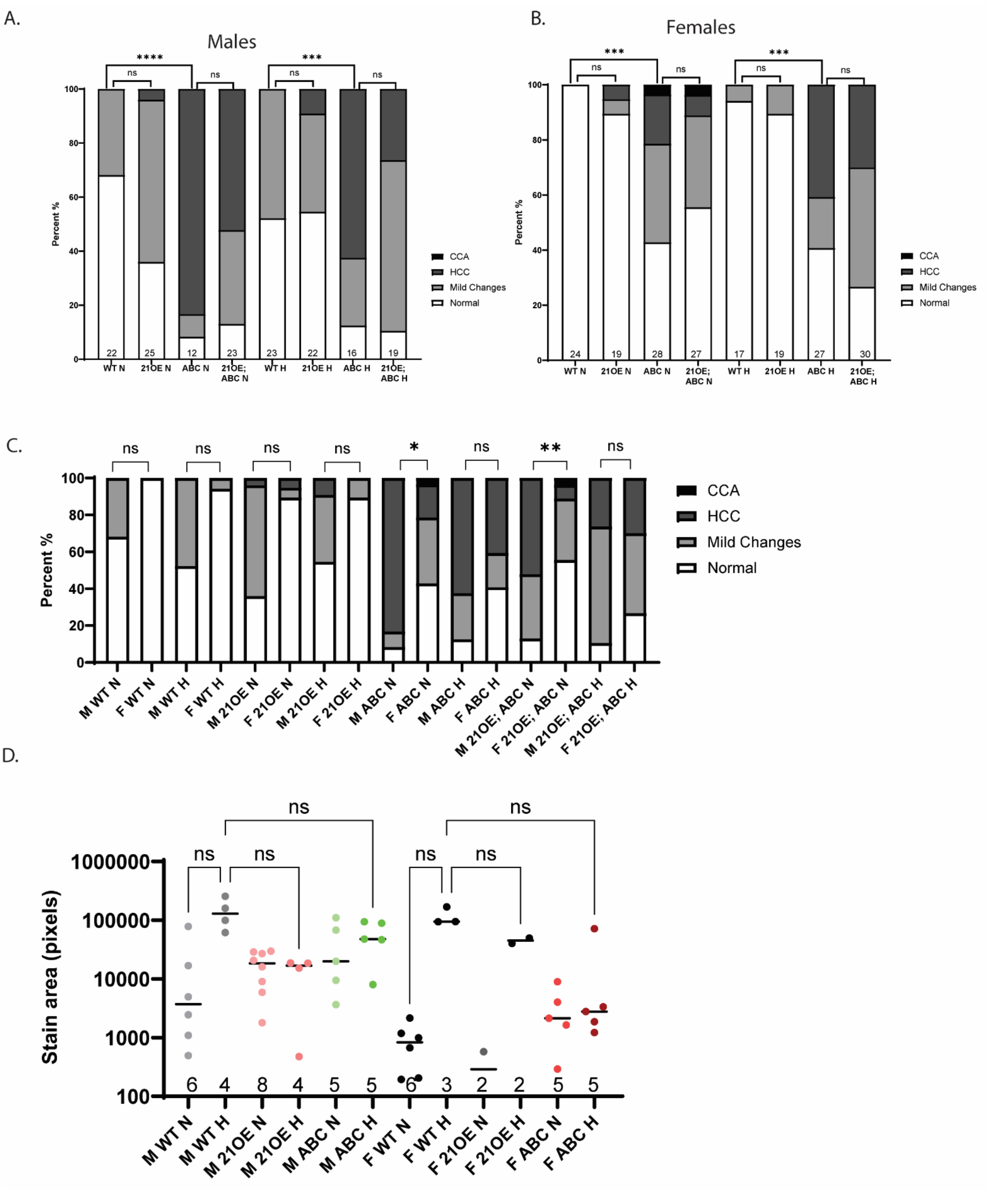

Disease Models & Mechanisms • Supplementary information

Fig. S17. Histologic changes by genotype, sex, and diet. Adult miR-21OE (21OE), ABC, and non-transgenic wild-type control (WT) sibling zebrafish were fed high cholesterol diet (H) or normal control diet (N). A-C. Histologic changes were scored as normal (0), mild changes (1), HCC (2), or cholangiocarcinoma (3) in a blinded fashion. These are the same data shown in Figure 4, but males (M) and females (F) were graphed and analyzed separately. P values determined with GraphPad Prism, Kruskal-Wallis test with Dunn's multiple comparisons test: ns, not significant; ***, $p < 0.001$; ****, $p < 0.0001$. For (C), all comparisons between high-cholesterol diet and normal control diet for the same sex and genotype were not statistically significant. D. Quantification of oil red O staining by sex. These are the same data shown in Figure 4, but males (M) and females (F) were analyzed separately. P values determined with GraphPad Prism, Brown-Forsythe and Welch ANOVA with Dunnett's T3 multiple comparisons test: ns, not significant.

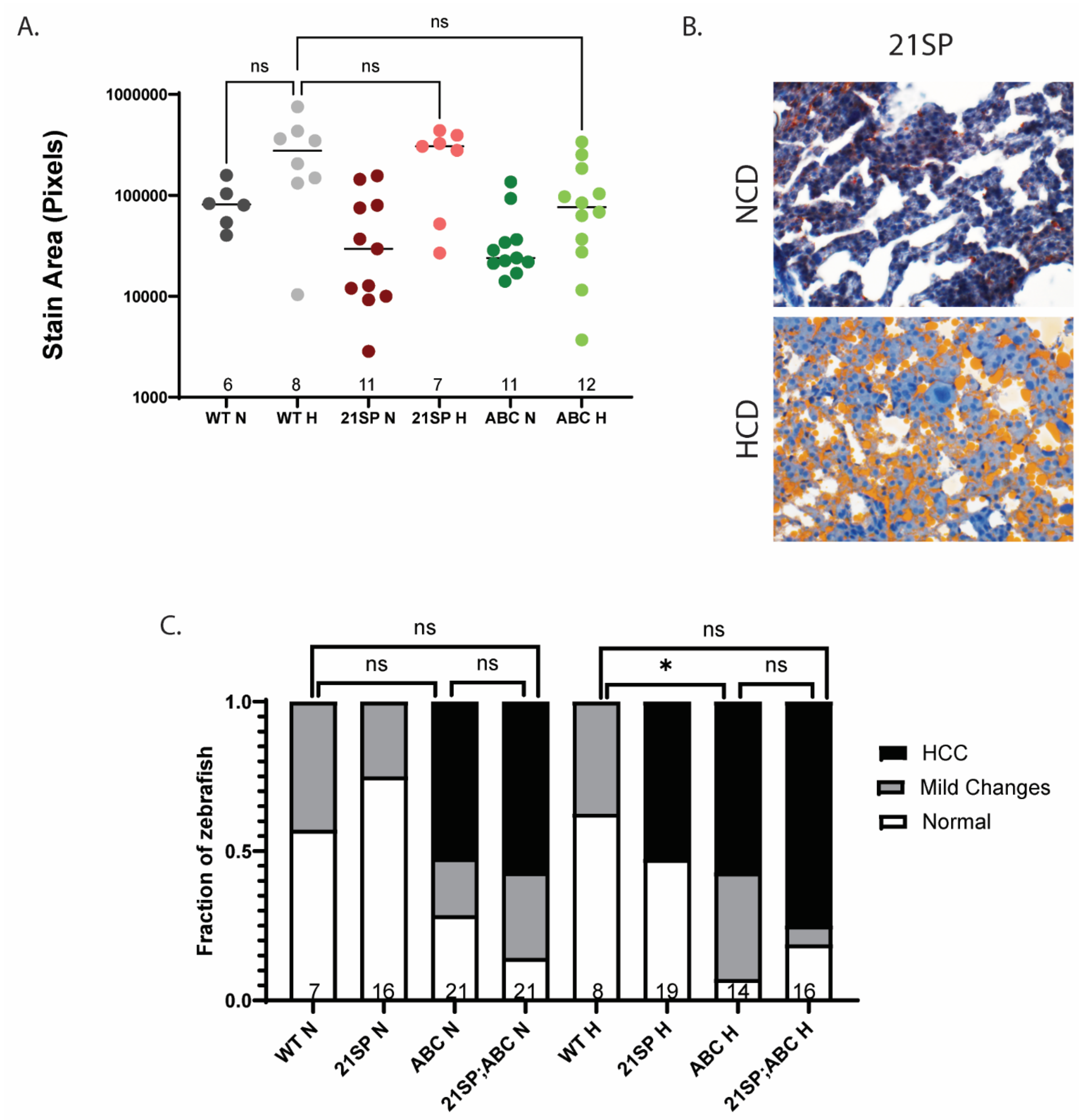

Fig. S18. miR-21SP does not affect steatosis or HCC incidence in adult zebrafish. A-C. We fed miR-21SP (21SP), ABC, miR-21SP; ABC double-transgenic (21SP;ABC), and non-transgenic wild-type control (WT) sibling zebrafish a high cholesterol diet (H, HCD) or normal control diet (N, NCD) as adults following scheme shown in Figure 4A. Quantification (A) and representative images (B) for hepatic oil red O staining revealed no significant difference in steatosis with miR-21SP compared to WT. Histologic analysis of zebrafish livers (C) showed no significant difference with miR-21SP, including in the presence of ABC. Three independent replicates were performed, and data from one representative experiment are shown. N values for each experiment are indicated at bottom of columns. P values determined with GraphPad Prism, Brown-Forsythe and Welch ANOVA with Dunnett's T3 multiple comparisons test (A) or Kruskal-Wallis with Dunn's multiple comparisons test: ns, not significant; *, $p < 0.05$.

Disease Models & Mechanisms • Supplementary information

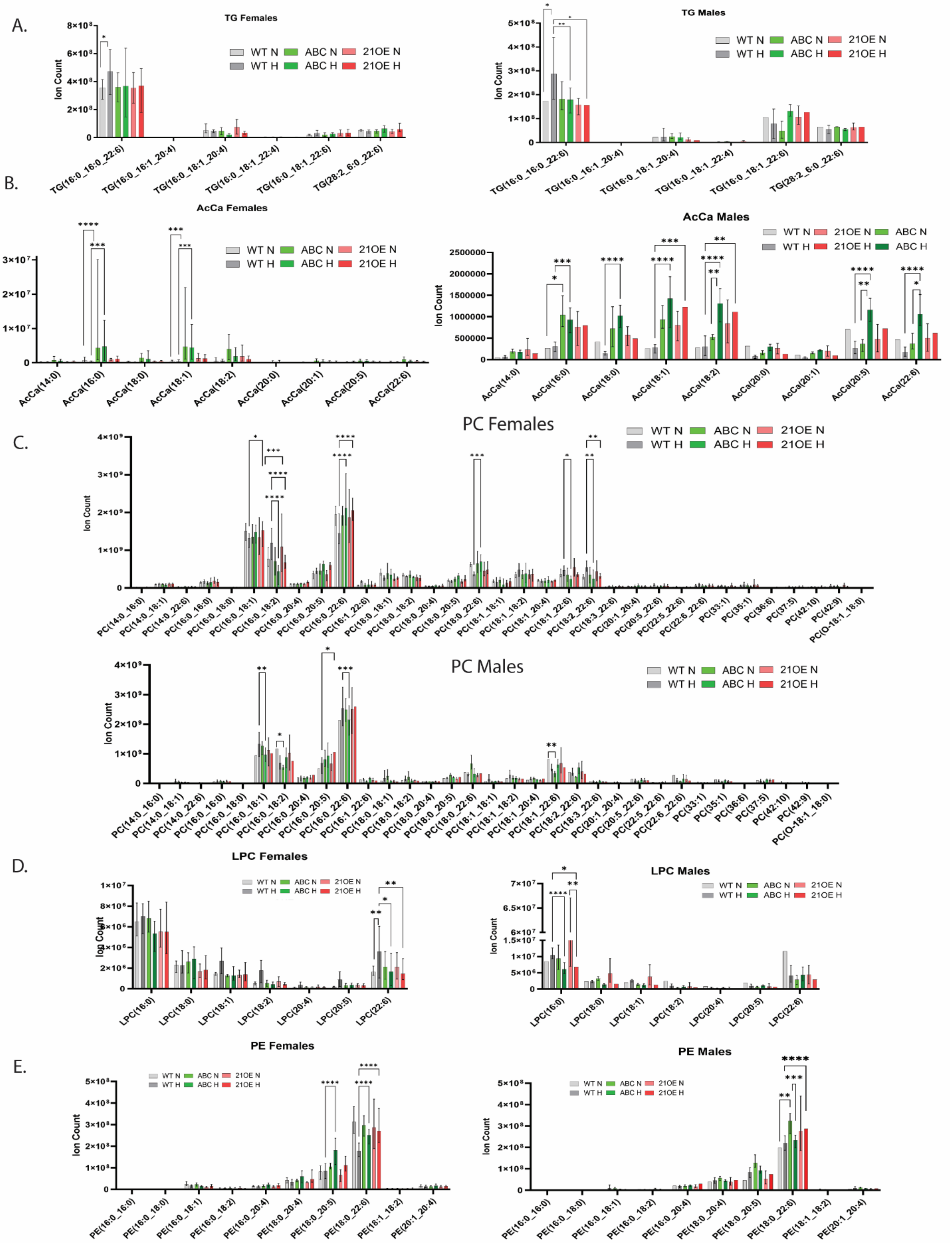

Disease Models & Mechanisms • Supplementary information

Fig. S19. Lipidomics analysis of male and female adult zebrafish. A. Triglyceride (TG) counts. B. Acylcarnitine (AcCa) counts. C. Phosphotidylcholine (PC) counts. D. Lysophosphotidylcholine (LPC) counts. E. Phosphotidylethanolamine (PE) counts. P values determined with GraphPad Prism, two-way ANOVA: *, < 0.05; **, p < 0.01; ***, p < 0.001; ****, p < 0.0001. All other comparisons were not statistically significant. N = normal cholesterol diet. H = high cholesterol diet. WT = non-transgenic wildtype sibling control. ABC = activated β-catenin overexpression. 21OE = miR-21 overexpression. Data is pooled from three independent experiments.

Table S1. Clinical and demographic information for patients with MASH or MASH-HCC

Patient	Diagnosis Category	Age	Sex	Race	Ethnicity	Diagnosis (tumor)	Diagnosis (non-tumor)	Other clinical diagnoses
1	Cirrhosis	37	M	White	Non-Hispanic	No malignancy	Cirrhosis	Morbid obesity, MASH
2	Cirrhosis	54	M	Other	Hispanic/ Latino/a/x	No malignancy	Cirrhosis	DM, MASH
3	Cirrhosis	44	M	Other	Hispanic/ Latino/a/x	No malignancy	Cirrhosis with mild steatosis	MASH
4	Cirrhosis	56	F	Mexican American	Hispanic/ Latino/a/x	No malignancy	Cirrhosis	MASH
5	Non-cirrhotic	68	M	Other	Hispanic/ Latino/a/x	Metastatic adenocarcinoma	Non-cirrhotic	Metastatic colorectal adenocarcinoma
6	Non-cirrhotic	69	M	White	Non-Hispanic	Metastatic adenocarcinoma	Non-cirrhotic, mild steatosis	Metastatic colorectal adenocarcinoma
7	Non-cirrhotic	69	M	White	Non-Hispanic	Hemorrhagic cavernous hemangioma	Non-cirrhotic	Complex hepatic cysts, high cholesterol, DM
8	Non-cirrhotic	61	F	White	Non-Hispanic	Metastatic adenocarcinoma	Non-cirrhotic	Metastatic colorectal adenocarcinoma
9	HCC+matched non-tumor	62	M	White	Non-Hispanic	HCC	Cirrhosis	MASH
10	HCC+matched non-tumor	74	M	White	Non-Hispanic	HCC	Cirrhosis	MASH, DM
11	HCC+matched non-tumor	68	M	White	Non-Hispanic	HCC	Cirrhosis	MASH
12	HCC+matched non-tumor	58	M	White	Hispanic/ Latino/a/x	HCC	Cirrhosis	Cryptogenic cirrhosis, no significant alcohol history, obesity, high cholesterol
13	HCC+matched non-tumor	69	M	White	Unknown	HCC	Cirrhosis	MASH, obesity, OSA
14	HCC+matched non-tumor	66	M	White	Non-Hispanic	HCC	Cirrhosis	Probable MASH, no significant alcohol history, obesity
15	HCC+matched non-tumor	68	F	White	Non-Hispanic	HCC	Cirrhosis	MASH, obesity

DM, Diabetes mellitus; OSA, obstructive sleep apnea

Disease Models & Mechanisms • Supplementary information

Table S2. miRNA analysis

Available for download at

https://journals.biologists.com/dmm/article-lookup/doi/10.1242/dmm.052583#supplementary-data

Table S3. miR-21 and HCD RNAsequencing results

Available for download at
https://journals.biologists.com/dmm/article-lookup/doi/10.1242/dmm.052583#supplementary-data

Table S4. Shiny Go

Available for download at
https://journals.biologists.com/dmm/article-lookup/doi/10.1242/dmm.052583#supplementary-data

Table S5. Zebrafish compared to DIO mouse

Available for download at

https://journals.biologists.com/dmm/article-lookup/doi/10.1242/dmm.052583#supplementary-data

Table S6. C3 TFT GSEA

Available for download at

https://journals.biologists.com/dmm/article-lookup/doi/10.1242/dmm.052583#supplementary-data

Table S7. miR21 and ABC ZF

Available for download at
https://journals.biologists.com/dmm/article-lookup/doi/10.1242/dmm.052583#supplementary-data

Disease Models & Mechanisms • Supplementary information

Table S8. Primers amplify miR-21. Primers to amplify *dre-mir-21-1* with BbsI cut sites.

Primer Name	Sequence
dre-mir-21-1_BbsI_F	*GTACGGGCTATGTCTTCccaccctctcctctcatcag*
dre-mir-21-1_BbsI_R	*GTACGGGCTATGTCTTCtggcgacgctaaaataagaca.*

Table S9. gBlock. IDT gBlockTM Sequence with MfeI and BamHI Cut Sites for Creation of Dendra2 Sponge. Bolded sequences highlight enzyme cut sites and the 6 sponge sites are underlined.

Sponge name	Sequence
dre-miR-21SP	*ctaATGAATG**CAATTG**TTGTTGTTgccaacaccctggataagctaGGAACCTTgccaacaccttggataagctaTCTGACGAgccaacacctatgataagctaCGCTTTAgccaacaccgtagataagctaGATCCTCGgccaacacctaggataagctaTATACCCGgccaacacctaagataagctaAACTTGTTTATTGCAGCTTATAATGGTTACAAATAAAGCAATAGCATCACAAATTTCACAAATAAAGCATTTTTTTCACTGCATTCTAGTTGTGGTTTGTCCAAACTCATCAATGTATCTTAAGGCGTAAATTGTAAGCGTTAATATTTTGTTAAAATTCGCGTTAAATTTTTGTTAAATCAGCTCATTTTTTAACCAATAGGCCGAAATCGGC**GGATCC**TGCGGCCatg*

Table S10. qPCR primers. Primer sequences for qPCR

Gene	Primer name	Sequence
Lactate dehydrogenase	LDHD FWD<br>LDHD REV	GCCTGGGGTGACACGAAAAA<br>GGACTGCATTTGTGCCTGATG
Ferroptosis suppression protein 1	FSP1 FWD<br>FSP1 REV	GGCTTTGACTTCATACAAAACAGAA<br>AAATGGAAGAGCGTGCAACC
Insulin-like growth factor (IGF) binding protein 2	igfbp2a FWD<br>igfbp2a REV	TGTGACAAGAGGGGGCAGTA<br>AGGGGCTTAAAGGGCAAGAG
Inter-Alpha-Trypsin Inhibitor Heavy Chain 2	Itih2 FWD<br>Itih2 REV	TGTGCAGATCCCCAAACGAG<br>CGTGCACTTCTGTCCGAAAC
vitamin D receptor	VDR FWD<br>VDR REV	GCTGATTAGCCTCGACTGTTCT<br>AATCCGGTGGCTTTGTCTCC
GABA Type A Receptor-Associated Protein	GABARAP FWD<br>GABARAP REV	AGGCGATCAGAGGGAGAGAA<br>CAGTCAGATCAGAAGGGACCAG
nicotinamide nucleotide transhydrogenase	nnt FWD<br>nnt REV	TTCCGGGTGACGGCTGTAAG<br>ACTAGTGAGGCGGTTGAGGG
Niemann-Pick disease type C2 protein	npc2 FWD<br>npc2 REV	CCACGCGTCCGAGGAAACT<br>AGTAAAACCACGCCGAGCA
Beta Actin	BACTIN FWD<br>BACTIN REV	CGAGCAGGAGATGGGAACC<br>CAACGGAAACGCTCATTGC
